# Pentatricopeptide repeat protein MID1 modulates *nad2* intron 1 splicing and *Arabidopsis* development

**DOI:** 10.1038/s41598-020-58495-5

**Published:** 2020-02-06

**Authors:** Peng Zhao, Fang Wang, Na Li, Dong-Qiao Shi, Wei-Cai Yang

**Affiliations:** 1 State Key Laboratory of Molecular and Developmental Biology, Institute of Genetics and Developmental Biology, Chinese Academy of Sciences, Beijing, 100101 China; 2 University of Chinese Academy of Sciences, Yuquan Road, Beijing, 10049 China

**Keywords:** Plant development, Seed development

## Abstract

As one of the best-studied RNA binding proteins in plant, pentatricopeptide repeats (PPRs) protein are mainly targeted to mitochondria and/or chloroplasts for RNA processing to regulate the biogenesis and function of the organelles, but its molecular mechanism and role in development remain to be further revealed. Here, we identified a mitochondria-localized P-type small PPR protein, MITOCHONDRION-MEDIATED GROWTH DEFECT 1 (MID1) that is crucial for *Arabidopsis* development. Mutation in *MID1* causes retarded embryo development and stunted plant growth with defects in cell expansion and proliferation. Molecular experiments showed that *MID1* is required for the splicing of the *nad2* intron 1 in mitochondria. Consistently, *mid1* plants display significant reduction in the abundance and activity of mitochondrial respiration complex I, accompanied by abnormal mitochondrial morphology and energy metabolism. Furthermore, MID1 is associated with other *trans*-factors involved in *NICOTINAMIDE ADENINE DINUCLEOTIDE HYDROGEN* (*NADH*) *DEHYDROGENASE SUBUNIT 2* (*nad2*) intron 1 splicing, and interacts directly with itself and MITOCHONDRIAL STABILITY FACTOR 1 (MTSF1). This suggests that MID1 most likely functions as a dimer for *nad2* intron 1 splicing. Together, we characterized a novel PPR protein MID1 for *nad2* intron 1 splicing.

## Introduction

Mitochondrion, originated from the gram-negative bacterium ancestors, is an essential organelle of eukaryotic organisms that can produce energy and signaling molecules for their growth and development^1–3^. Most of the mitochondrial genes have been transferred and integrated into nuclear genome during evolutionary progress, and the remaining genes encode a limited number of proteins that mainly make up the respiration complexes and translation machinery^4–7^. Thus, mitochondria are semi-autonomous organelles that require pathways to coordinate the two genomes for biogenesis and function^8,9^. Among the pathways controlling mitochondrial genes expression are RNA binding proteins-mediated post-transcriptional events in mitochondria such as RNA stability, RNA editing, intron splicing and translation^10–13^. Genetic studies have shown that these molecular events are critical for mitochondrial function and plant development^11,14^. Although the molecular function of many mitochondrion-localized RNA binding proteins has been characterized, their precise molecular action remains to be further unveiled.

In plants, most of the well-characterized RNA-binding proteins involved in post-transcriptional regulation of mitochondrial genes are PPR proteins, which are defined by tandem arrays of degenerate 35 amino acid motif^11,15^. PPR proteins are first discovered by *in silico* study of incomplete *Arabidopsis* genome for genes targeting to mitochondrion and chloroplast^16^. The striking feature of this family is its massive expansion in terrestrial plants, about 450 members in *Arabidopsis* and 477 members in rice^17,18^. These proteins can be divided into two groups based on the PPR motif length: P-type and PLS-type, and further into several subgroups based on the C-terminal characteristics^17,19^. The P-type PPR proteins only consist of the 35-amino-acid classic PPR (P) motifs, while the PLS-type ones have P and longer (L) or shorter (S) PPR motifs, with extra domains at the carboxyl terminus. Consistent with the initial bioinformatics study, the majority of studied PPR proteins are localized to mitochondria and/or chloroplasts, where they usually function in diverse RNA metabolic processes including intron splicing, editing, stabilization and translation^11^. Moreover, the activity of PPR proteins, especially their binding codes, have been progressively elucidated through bioinformatics and structural analysis^20–26^. Similar to classic RNA-binding protein Pumilio and FBF (PUF)^27^, PPR proteins bind RNA targets in a modular fashion, with each PPR motif specifically recognizing one nucleoside and two amino acids at the 5th and 35th of the PPR motif primarily determining the nucleoside specificity^11,24^. Although great advances have been made on understanding PPR codes, the action mode of PPR proteins is uncertain. Some PPR proteins were reported to play roles in a dimer manner, such as HIGH CHLOROPHYLLFLUORESCENCE 152 (HCF152) and THYLAKOID ASSEMBLY 8 (THA8), two chloroplast-localized PPR proteins involved in RNA processing and splicing respectively^28–30^. PPR4 and PPR5 are two examples that they exist as monomers to function in *rps12 trans*-splicing and stability of t-RNA precursor in chloroplasts^31,32^. However, PPR10, one of best characterized PPR proteins, is still in debate that whether it acts in a dimer or monomer manner to perform function^24,33–35^.

RNA splicing, a common event taking place in nucleus and organelles of eukaryotic organisms, is a necessary step in post-transcriptional regulation of gene expression^36^. While the constituents and action mode of spliceosome that splices intron in the nucleus have been extensively studied^37^, little is known about the splicing machinery responsible for excision of mitochondrial introns. Previous genetic studies have led to the identification of several P-type PPR proteins as necessary factors involved in mitochondrial intron splicing in model species *Arabidopsis thaliana* and *Zea mays*. For example, ABA OVERLY SENSITIVE 5 (ABO5), BUTHIONINE SULFOXIMINE (BSO)-INSENSITIVE ROOT 6 (BIR6), MITOCHONDRIAL INTRON SPLICING FACTOR 26 (MISF26), MISF74, MITOCHONDRIAL TRANSLATION FACTOR1 (MTL1), ORGANELLE TRANSCRIPT PROCESSING 43 (OTP43), OTP439, TANG2, SLOW GROWTH3 (SLO3), are required for splicing of some introns of *nad* genes in *Arabidopsis*^38–45^. Interestingly, MISF68 and MISF74 are two P-type PPR proteins reported to function in more than one introns splicing in mitochondria^45^. EMPTY PERICARP 10 (EMP10), EMP11, EMP16 and DEFECTIVE KERNEL (DEK) 2, DEK35, are involved in the splicing of some introns of *nads* in maize^44,46–49^. Besides, some PLS-type PPR proteins which are usually characterized to function in RNA editing, are also implicated in RNA splicing. SLO4 is reported to affect *nad2* intron 1 splicing^50^. A PLS-DYW member, PpPPR43 influences the *CYTOCHROME C OXIDASE 1*(*cox1*) intron 3 splicing in *Physcomitrella patens*^51^. Furthermore, other *trans*-factors implicated in RNA splicing have also been identified, such as CHLOROPLAST RNA SPLICING AND RIBOSOME MATURATION (CRM) proteins, PLANT ORGANELLAR RNA RECOGNITION (PORR) proteins, DEAD-BOX RNA-HELICASEs, MITOCHONDRIAL TRANSCRIPTION TERMINATION FACTORs (mTERFs), REGULATOR of CHROMOSOME CONDENSATION (RCC) proteins, RAD52-LIKE PROTEINS AND NUCLEAR MATURASEs (nMATs)^41,52–59^. How PPR proteins cooperate with these *trans*-factors to promote introns splicing is not clear. Deciphering the relationship among the previously found splicing factors and identifying their RNA-binding sites will be of great significance to understand the molecular mechanism underlying the mitochondrial RNA splicing.

In this study, we isolated a mutant defective in embryo development and vegetative growth, designated *mitochondrion-mediated growth* *defect 1* (*mid1*) according to its molecular and physiological function. We showed here that *MID1* encodes a mitochondria-localized PPR protein with only four PPR motifs and is responsible for the splicing of intron 1 in the *nad2* gene. Loss of *MID1* function severely impairs the abundance and activity of mitochondrial respiration complex I, which further leads to the abnormal mitochondrial morphology and energy metabolism. In addition, MID1 is associated with other *trans*-factors to function in *nad2* splicing. Together, we propose that MID1-mediated RNA splicing is necessary for mitochondrion biogenesis and function, and further plays a critical role in plant development.

## Results

### Isolation of embryo-defective mutants

To isolate embryo-defective mutants, a distorted Mendelian segregation screen was performed as previously reported^60,61^. *mid1* was one of the mutants in which the kanamycin-resistant to kanamycin-sensitive separation ratio of selfed F_2_ progeny was 2.63:1 (n = 781), indicating that there was a minor deficiency in *mid1* embryogenesis^62^. The reciprocal crosses between *mid1*/+ and wild-type plants were subsequently conducted to further confirm this speculation. When the pistil of the wild-type was pollinated with pollen from *mid1*/+ plant, the segregation ratio was 0.91:1(n = 410), and *vice versa* (0.98:1, n = 1306). The ratio was close to the expected 1:1, indicating that the *Ds* insertion did not disrupt the male and female gametophyte function. Thus, *mid1* is most likely an embryo-defective mutant.

### *MID1* is required for embryonic and post-embryonic development

**Figure 1:**
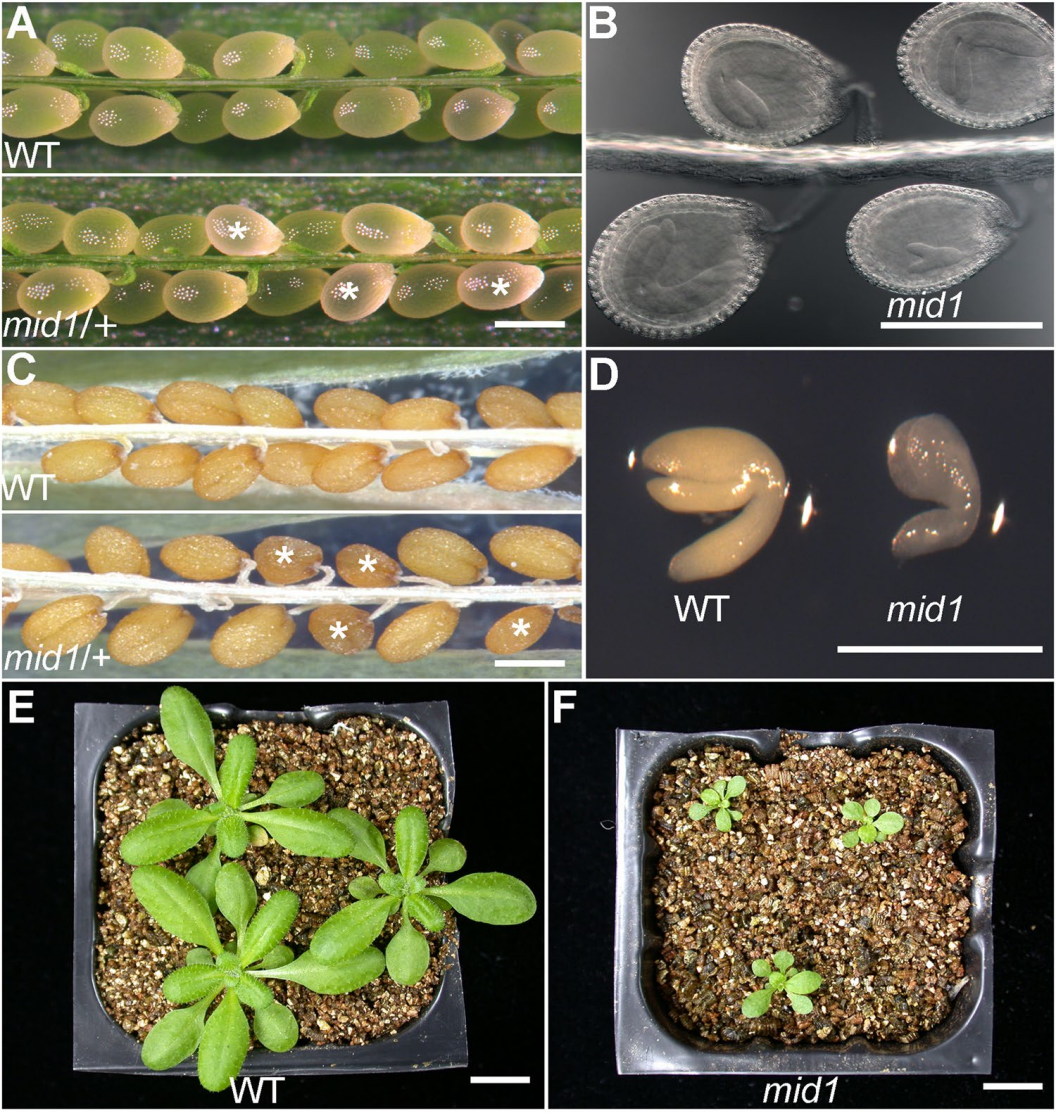
Phenotypes of *mid1*. (**A**) Seed set of wild-type and *mid1*/+ siliques at 8 DPP. The wild-type shows a full seed set while approximately one-quarter of ovules in the *mid1*/+ silique are abnormal. Asterisks indicate the *mid1* ovules. (**B**) Whole-mounted ovules from silique of *mid1*/+ plant in (**A**). (**C**) Seeds in the mature silique of wild-type and *mid1*/+. Compared to the wild-type, about one-quarter of the seeds in *mid1*/+ display shrunken morphology. (**D**) Embryos dissected from *mid1*/+ plant. (**E**) 4-week-old wild-type plants. (**F**) 4-week-old *mid1* plants. (**A**,**F**) Bars = 200 μm; (**E**,**F**) Bars = 1 cm.

To investigate the embryo phenotype, we dissected siliques of wild-type and *mid1*/+ plants, followed by whole-mount clearing and elaborate microscopic examination. Compared to the synchronous development of wild-type embryos (Fig. 1A), there was a significant developmental discrepancy in embryos of *mid1*/+ siliques (Fig. 1A,B). For example, at 8-days post pollination (DPP), the wild-type embryos were all at curled cotyledon stage, while about 25% embryos (195:790) of *mid1*/+ were still at torpedo stage (Fig. 1B). However, the mutant embryos in the *mid1*/+ were able to reach mature stage, and finally form small brown shrunk seeds harboring small but intact embryos (Fig. 1C,D), indicating that *mid1* caused retarded embryogenesis. In addition to the delayed embryogenesis described above, *mid1* also impairs the post-embryonic development, characterized by stunted plant growth. For example, the 4-week-old *mid1* plant was much smaller than the wild-type (Fig. 1E,F). These results demonstrate that *MID1* is crucial for both embryonic and post-embryonic development in *Arabidopsis*.

### Cell expansion and proliferation are compromised in *mid1*

**Figure 2:**
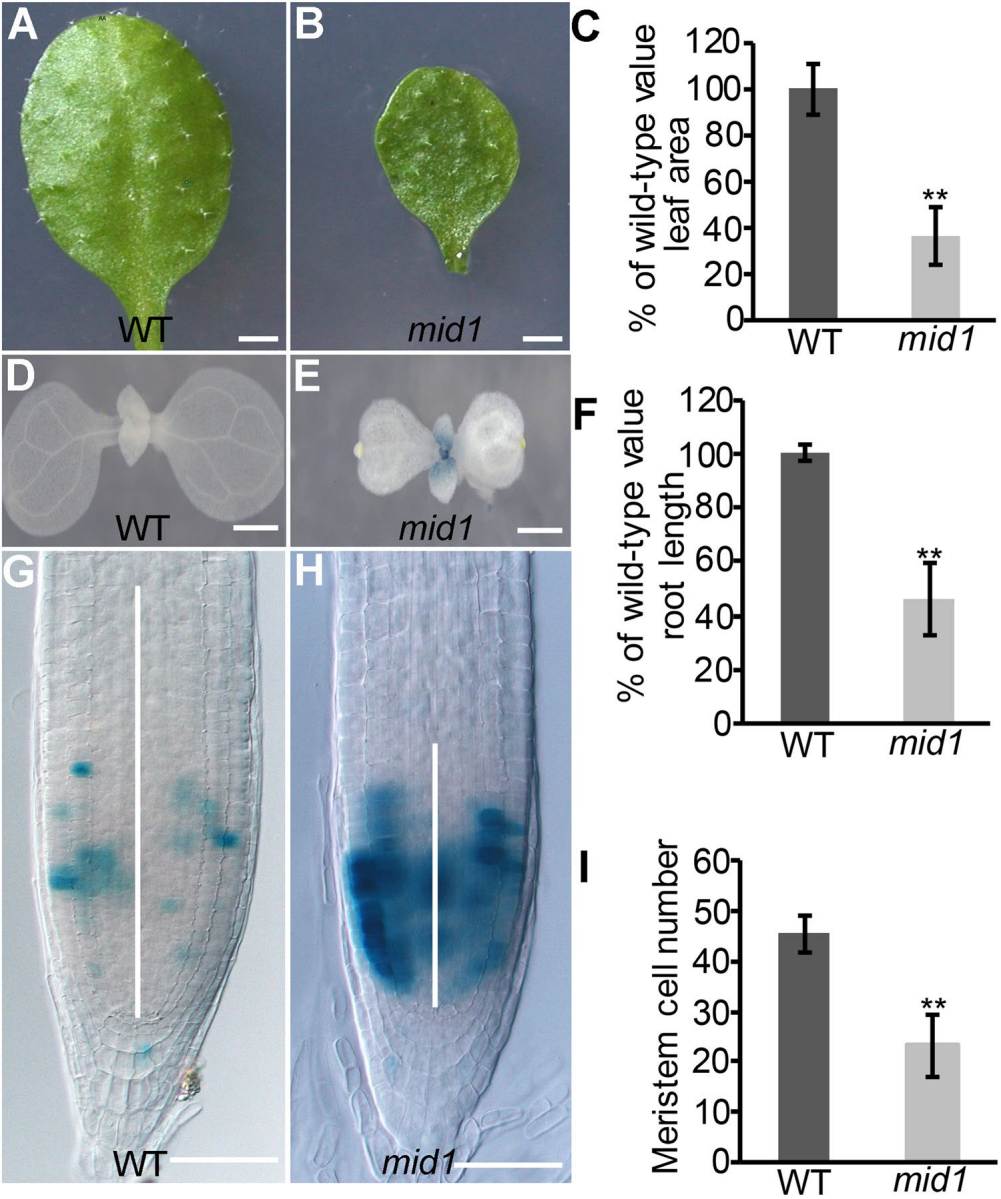
Cellular defects of *mid1*. (**A**) Fifth leaf of 4-week-old wild-type plant. Bar = 0.3 cm. (**B**) Fifth leaf of 4-week-old *mid1* plant. Bar = 0.3 cm. (**C**) Leaf area measurement of wild-type and *mid1*. Data are the mean ± SE; n = 35. Student’s t tests were used to analyze differences between WT and *mid1*. Double asterisks represent highly significant differences, **P < 0.01. (**D**) Distribution of CYCB1;1-GUS in the 8-day-old wild-type true leaf. Bar = 1 mm. (**E**) Distribution of CYCB1;1-GUS in the 8-day-old *mid1* true leaf. Bar = 1 mm. (**F**) Primary root length of 8-day-old wild-type and *mid1* seedlings. Data are the mean ± SE, n = 40. Student’s t tests, **P < 0.01. (**G**,**H**) Expression of CYCB1;1-GUS in the root meristem of 8-day-old wild-type and *mid1*. The white lines indicate the meristem zone. Bars = 50 μm. (**I**) Root meristem cells number of 8-day-old wild-type and *mid1* seedlings. The root meristem cells are defined by the number of cortex cells in the cortical file extending from the QC to the transition zone. Data are the mean ± SE, n = 40. Student’s t tests, **P < 0.01.

One of the distinct phenotypes of *mid1* is its dwarfism morphology during the whole life cycle, such as small seed, small leaf and stunted root. Previous studies have indicated that cell proliferation and expansion are the main determinants controlling organ size^63^. In order to confirm the cellular basis responsible for the smallness, we checked the capacity of cell expansion and proliferation in diverse organs of *mid1*. In *Arabidopsis*, the fifth leaf is the first fully developed leaf and widely used as the representative organ to assess cell expansion capacity^63^. First, we found that the fifth leaf of *mid1* was much smaller than the wild-type (Fig. 2A–C). Then, we investigated the cell area of the fifth leaf to determine whether the cell size contribute to the small leaf size. The *mid1* leaf cell area was dramatically decreased, about 30% of the wild-type counterpart (Fig. S1A). Second, we investigated *mid1* cell proliferation capacity by monitoring the mitotic index through introducing the mitotic activity reporter CYCB1;1-GUS (for β-glucuronidase) into *mid1*^64^. Indeed, histochemical staining of 8-day-old seedling showed that CYCB1;1-GUS was expressed in young leaves of *mid1* while nearly no GUS expression was detected in wild-type plant (Fig. 2D,E), a evidence that many leaf cells in *mid1* were retarded at G2/M phase, thus implying that *mid1* is deficient in leaf cell proliferation. Next, we also evaluated the effect of *MID1* mutation on the root development. Similarly, the root of *mid1* seedling was short (Fig. 2F) and there were more root meristem cells expressing CYCB1;1-GUS of 8-day-old *mid1* seedlings compared to the wild-type (Fig. 2G,H), indicating that loss-function of *MID1* also impaired the root cell proliferation in roots. Consistently, the root meristem cell size, determined by the cortical cell number in the region from quiescent center to the first elongation cell^65,66^, was greatly reduced in *mid1* (Fig. 2I). To check whether cell expansion was also altered in *mid1* roots, we measured cell length at the maturation region and found that the *mid1* cell length was dramatically shorter than that of the wild-type, suggesting that root cell expansion was also impaired in *mid1* root (Fig. S1B).

Furthermore, we performed the callus induction experiments to confirm the causal relationship between *mid1* phenotypes and cell proliferation deficiency. The primary roots of *mid1* and wild-type seedlings were first cut into segments of 0.5 cm in length, followed by 10 days culturing on Murashige and Skoog (MS) medium in the presence of 100 μg/L of 2,4-D and variable amounts of kinetin. As shown in Fig. S2A, callus from the wild-type plant proliferated rapidly at the indicated concentration of hormone, with a tendency that callus growing better following the increment of kinetin. In contrast, the proliferation of *mid1* callus was less robust, even displaying growth repression at high concentration of kinetin. Similarly, the cotyledon explants of *mid1* grew slowly compared to the wild-type (Fig. S2B). Collectively, we concluded that mutation of *MID1* disrupts, most likely indirectly, the cell proliferation of diverse organs.

### Molecular cloning of *MID1*

**Figure 3:**
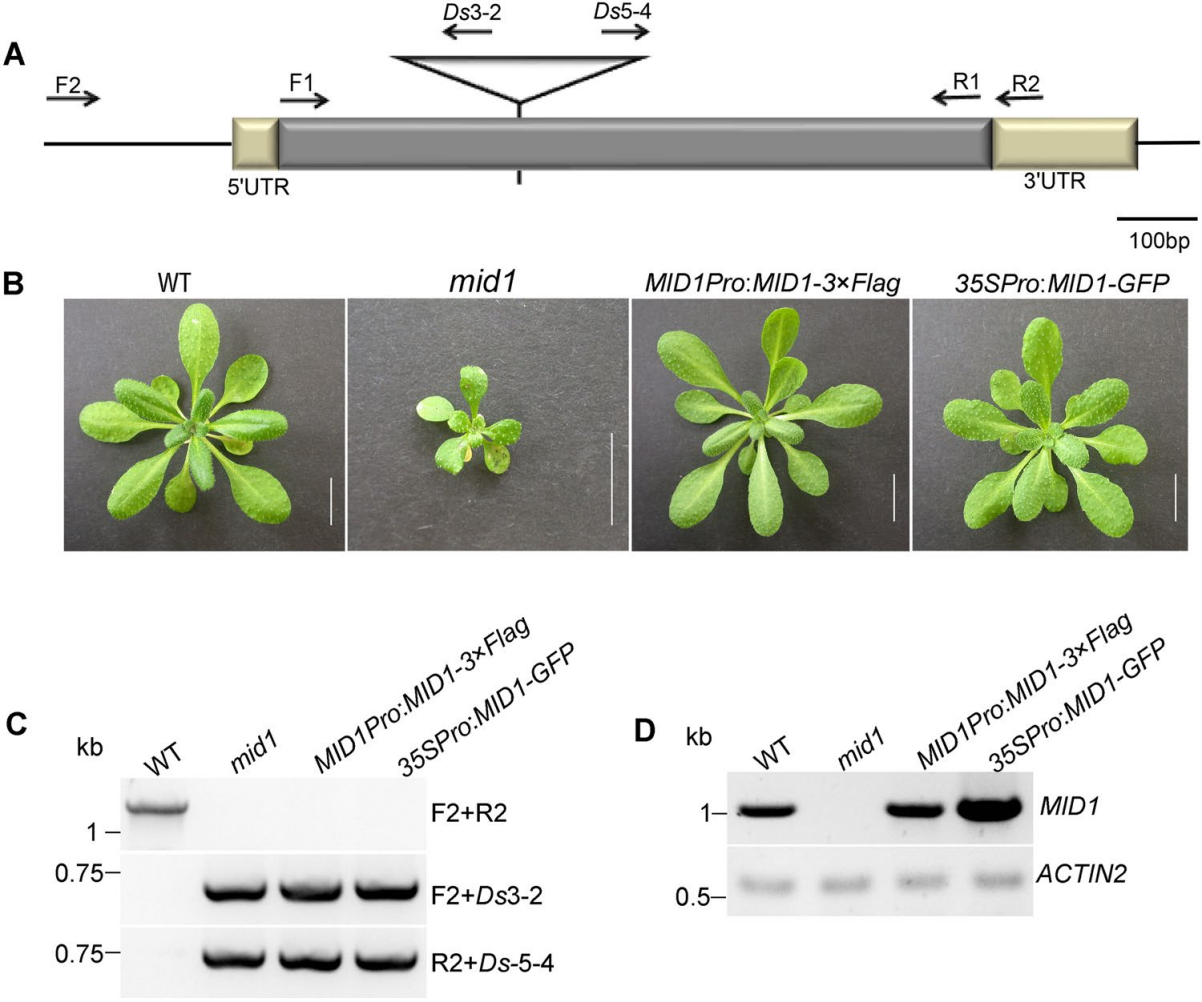
Cloning of *MID1*. (**A**) Diagram of *mid1* mutant. The *Ds* insertion is pointed out by a triangle. The position of primers used in (**C**,**D**) are displayed by arrows. (**B**) The phenotype of 4-week-old wild-type (WT), *mid1*, and genetically complemented lines *MID1Pro*:*MID1*-*3* × *Flag* and *35SPro*:*MID1*-*GFP*. Bar = 1 cm. (**C**) Genotyping of plants in (**B**) using the primers indicated in (**A**). (**D**) RT-PCR detection of *MID1* cDNA in plants (**B**) to confirm the successful complementation. The primers F1 and R1 were used to RT-PCR analysis of *MID1*.

To clone the *MID1* gene, thermal asymmetric interlaced PCR (TAIL-PCR) was employed to obtain the genomic sequence flanking the *Ds* element^67^. Sequence analysis indicated that the *Ds* was inserted into the *At1g06270*, at 342 bp downstream of ATG codon, generating 8 bp duplication at the insertion site (Fig. 3A). Meanwhile, we generate a CRISPR/Cas9-derived *mid1* mutant in Col-0 background, where a single A was inserted at 106 bp, causing frameshift and further resulting in premature stop codon after 6 erroneous codons^68^ (Fig. S3A). This mutant was named *mid1-2* while the mutant in L*er* background was named *mid1-1*. Phenotype analysis clearly displayed *mid1-2* was similar with *mid1-1*, such as retarded embryogenesis (Fig. S3B–E), small seed with intact embryo (Fig. S3F–I), and dwarfism at adult stage (Fig. 3J,K). The subsequent genetic and functional analysis mainly focus on *mid1-1*. The fragment containing the coding sequence of *MID1* under the control of either its native promoter (1053 bps upstream of initial ATG codon) or cauliflower mosaic virus promoter (CaMV 35S) could complement the mutant phenotype of *mid1-1* (Fig. 3B,C). Therefore, these findings demonstrate that the loss-function of *MID1* is indeed the cause of the altered developmental phenotypes of *mid1-1* plants. Besides, we failed to detect any *MID1* transcripts in *mid1-1* plants, suggesting that *mid1-1* was a null allele mutant (Fig. 3D).

*MID1* contains an open reading frame of 1032 bp that encodes a 40 KD protein with 343 amino acid (aa) residues. Different PPR protein predictors identify different numbers of PPR motif in MID1. For example, plantPPR and TIGRFAM annotates four PPR motifs in MID1, while PROSITE annotates five PPR motifs^69^, and UNIPROT annotates six PPR motifs^70^. The discrepancy between these predictors arises from the position of first PPR motif. plantPPR annotates 136^th^–170^th^ amino acids as the first PPR motif, TIGRFAM annotates 101^th^–135^th^ amino acids as the first PPR motif^19,71^, We adopt the annotation from plantPPR due to that it is a recently developed tool that redefines the PPR motif and have more accurate and consistent annotations of PPR sequences^19^. As annotated from plantPPR database (http://www.plantppr.com)^19^, MID1 is a member of the P-type PPR family, containing four putative canonical PPR motifs between amino acid residue 136^th^ and 311^th^. A BLAST search with MID1 protein sequence identified several orthologues from many eudicot plants (Fig. S4). Protein alignment showed that they share high similarity at protein level (Fig. S5). However, there is no close orthologues in monocots, indicating that MID1 is a novel P-type PPR protein unique to eudicots.

### *MID1* encodes a mitochondria-localized PPR protein

**Figure 4:**
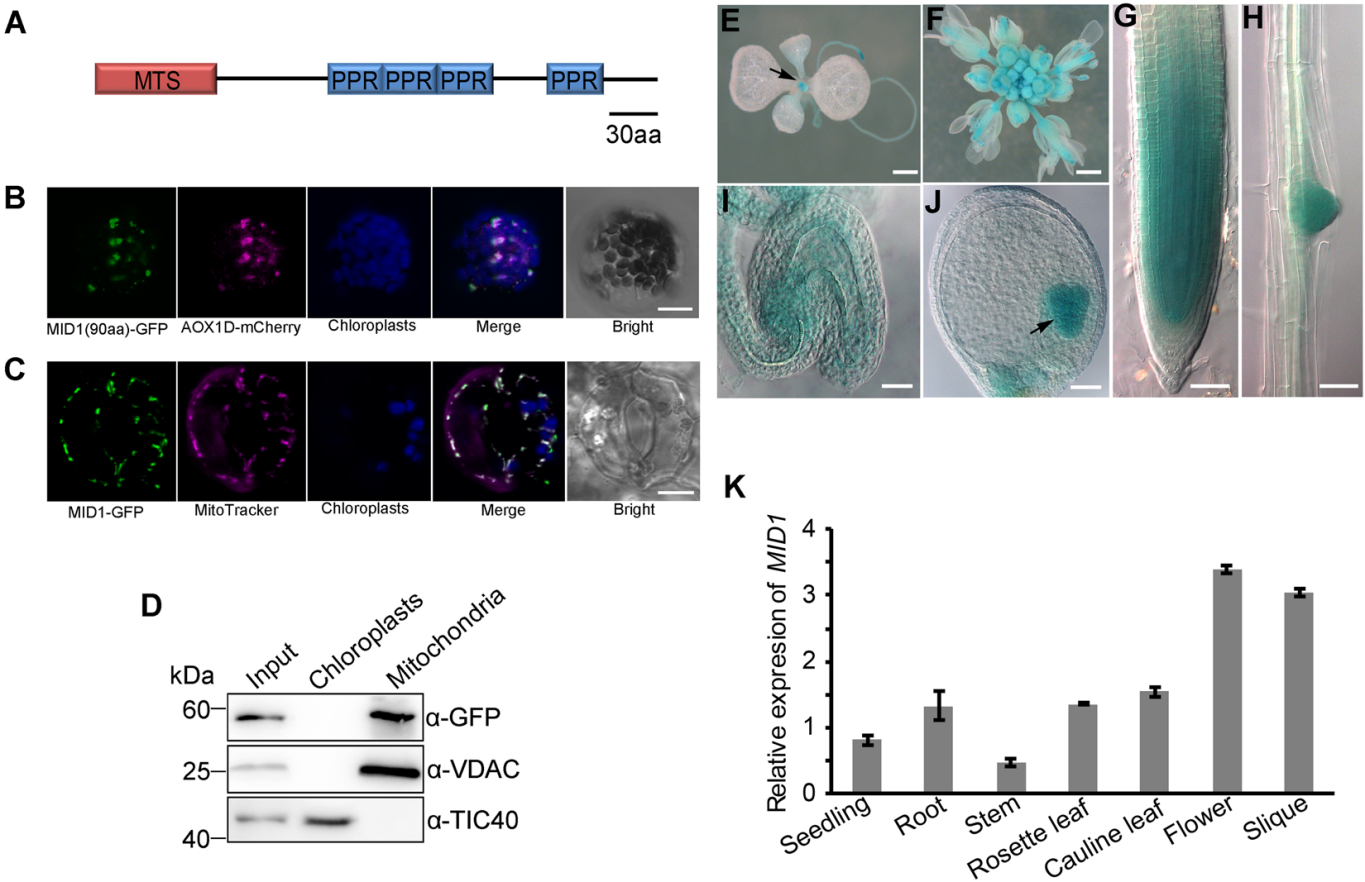
The subcellular localization and expression pattern of MID1. (**A**) Schematic structure of MID1. MTS, mitochondrial transient signal. (**B**) Subcellular localization of MID1-GFP in *Arabidopsis* protoplasts. Bar = 10 μm. (**C**) Subcellular localization of MID1-GFP in the stomata of *35SPro*:*MID1*-*GFP* transgenic seedling. Bar = 5 μm. (**D**) Immunodetection of MID1-GFP localization. α-VDAC and α-TIC40 are mitochondrion and chloroplast-specific antibodies, respectively. (**E**–**I**) The expression pattern of MID1-GUS reporter. (**E**) seedling, arrow indicates shoot apex, (**F**) flower, (**G**) root meristem, (**H**) lateral root primordium, (**I**) unfertilized ovule and (**J**) ovule with heart embryo, arrow indicate the embryo. (**E**,**F**) Bars = 1 mm, (**G**–**J**), Bars = 50 μm. (**K**) Relative expression levels of *MID1* mRNA in diverse tissues measured by qRT-PCR. The mean data are derived from triple independent biological replicates prepared from three parallel samples, and finally normalized with *ACTIN2*.

Both the Predotar and TargetP programs predicted that MID1 is a mitochondrial protein with the N-terminal 76 aa as a putative transit peptide targeted to mitochondrion^72–74^ (Fig. 4A). To determine its subcellular localization experimentally, the fusion containing the N-terminal of MID1 and GFP reporter was generated and transiently expressed in *Arabidopsis* protoplasts. Meanwhile, AOX1D, a mitochondria-localized alternative oxidase, was used as a mitochondrion marker. Confocal microscopy showed that the GFP signal was localized in mitochondria, but not in chloroplasts (Fig. 4B). Then we transferred *35SPro*:*MID1*-*GFP* into *mid1-1* mutant plants. The results showed that *35SPro*:*MID1*-*GFP* fully complemented the *mid1-1* mutation (Fig. 3B), suggesting that *MID1-GFP* was functional. We further checked the MID1 localization in the complemented plants. MID1-GFP was also localized in mitochondria as revealed by MitoTracker staining and there was no co-localization with chloroplasts (Fig. 4C). Besides, we separately extracted mitochondria and chloroplasts from the complemented lines, followed by Western blot detection. The result clearly showed that MID-GFP was enriched in mitochondria, not in chloroplasts (Fig. 4D). Together, these data confirm that *MID1* encodes a mitochondria-localized protein.

In order to get a better understanding about the function of *MID1* during development, we next investigated its temporal and spatial expression. First, we constructed a *MID1Pro:MID1*-*GUS* fusion under the control of its own promoter, which was subsequently transformed into the *mid1−1*/+ plants. With the help of the double antibiotic selection, the transgenic lines containing both *MID1Pro:MID1-GUS* and *mid1* were identified from the F_3_ progeny. In these plants, strong GUS signal was detected in multiple organs demanding for high cell proliferation activity, which include the shoot apex (Fig. 4E), flower (Fig. 4F), root meristem (Fig. 4G), lateral root primordium (Fig. 4H), unfertilized ovule (Fig. 4I) and embryo (Fig. 4J). By contrast, there was nearly no GUS activity in the cotyledon and root maturation region where the cell proliferation activity is low (Fig. 4E,H). In general, *MID1* is preferentially expressed in tissues with active cell division. To corroborate the expression pattern of *MID1*, the quantitative RT-PCR (qRT-PCR) experiments were employed to detect the relative *MID1* mRNA abundance in major organs of *Arabidopsis*. *MID1* was ubiquitously expressed in the organs tested, with high expression in reproductive organs such as flower and silique (Fig. 4I,K), which were consistent with the GUS staining results.

### MID1 is required for the splicing of *nad2* intron 1 and mitochondrial biogenesis

**Figure 5:**
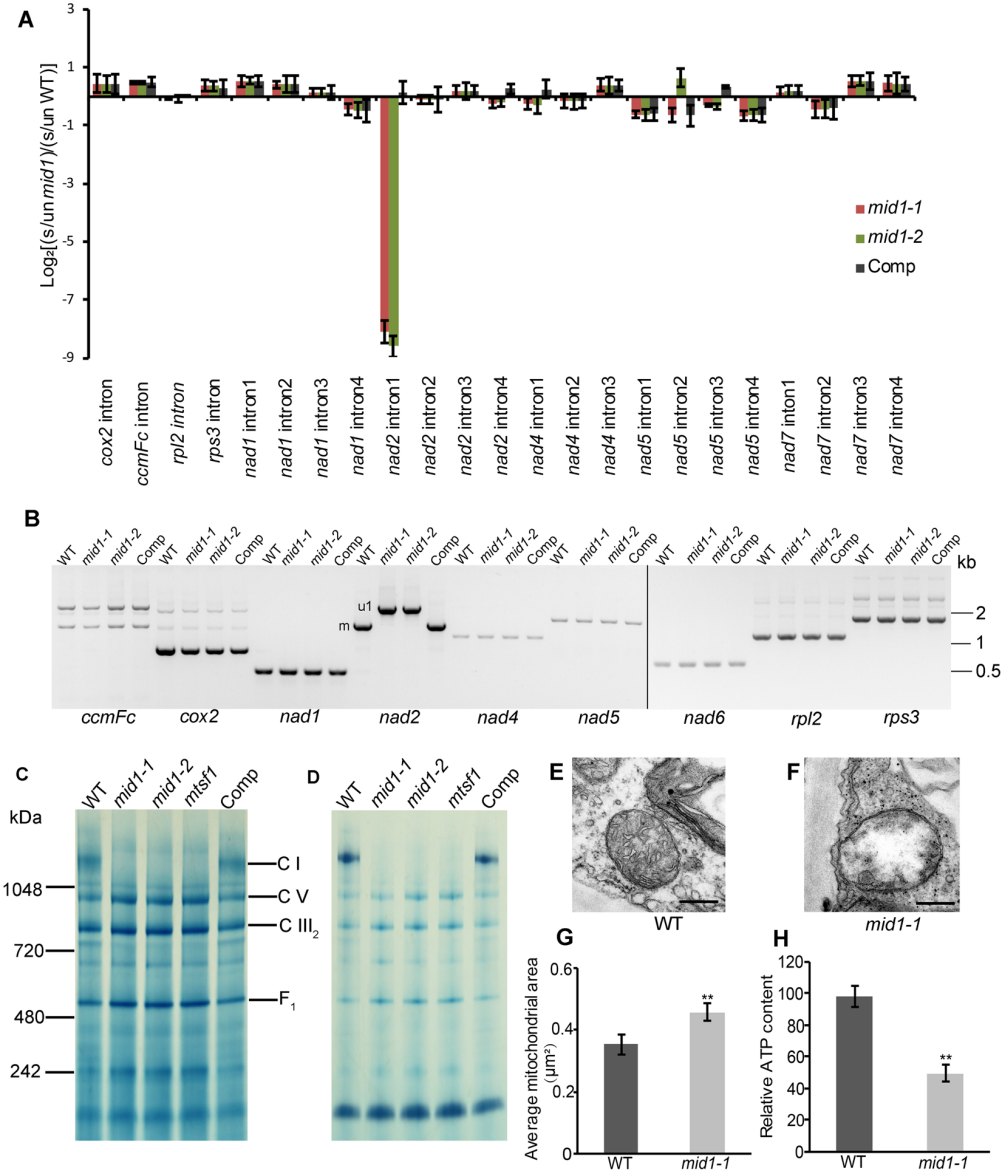
Functional analysis of *MID1* in mitochondria. (**A**) qRT-PCR analysis of splicing efficiency of mitochondrial introns in *mid1*. The histograms display the ratio of the spliced transcripts to the unspliced transcripts in *mid1* plants as compared with wild-type in a log_2_ form. Each mean value is the average of three biological replicates, each of which undergoes triple repeats. s, spliced transcripts; un, unspliced transcripts. (**B**) RT-PCR analysis of mitochondrial transcripts splicing efficiency. u1 means *nad2* transcript with intron 1.m means mature *nad2* transcript. comp, *MID1Pro:MID1*-3 × *Flag* complemented line. (**C**) BN-PAGE analysis of mitochondrial protein complexes abundance in wild-type, *mid1* mutants, *mtsf-1* and complemented line. Protein marks are indicated in the left panel and the bands representing main protein complexes are listed in the right panel. (**D**) In-gel analysis of complex I activity in wild-type, *mid1* mutants, *mtsf1* and complemented line. comp, *MID1Pro:MID1*-3 × *Flag* complemented line. Transmission electron microscopy analysis of mitochondrial ultrastructure in leaf of 4-week-old WT (**E**) and *mid1-1* (**F**). Bars = 0.5 μm. (**G**) Mitochondrial area of wild-type and *mid1-1*. Data are the mean ± SE, n = 140. Student’s t tests, *P < 0.05. (**H**) ATP content in the leaf of wild-type and *mid1-1*, Student’s t tests, **P < 0.01.

As we have noted, *MID1* encodes a PPR protein belonging to P-type subfamily, many of which were shown to be involved in RNA splicing within organelles^29,38–40^. So, it is reasonable to speculate that *MID1* is potentially involved in mitochondrial RNA splicing. Hence, we detected the splicing efficiency of 23 mitochondrial group II introns in 4-week-old wild-type, *mid1-1,mid1-2* and complemented plants using qRT-PCR^38,75^. The results showed that the splicing efficiency of *nad2* intron 1 is dramatically altered while there was no significant alternation in other introns, which suggest that *mid1* is defective in the splicing of *nad2* intron 1 (Fig. 5A). We further confirmed this RNA splicing deficiency by RT-PCR. First, as shown in Fig. 5B, the precursor *nad2* RNA with the intron 1 accumulated in *mid1* mutants, and the mature *nad2* without intron 1 was restored in the complemented line. Second, consistent with the qRT-PCR analysis, there were no splicing differences between the wild-type and *mid1* mutants regarding to other mitochondrial transcripts with introns. Furthermore, we also detected the editing status of *nad2* in *mid1* mutants to test whether MID1 is involved in *nad2* editing. 31 C-U editing events were reported to take place in *nad2*^76^, but only 27 editing sites were found in either WT mature *nad2* or *mid1 nad2* with intron 1 in our analysis. Among the identified editing sites, only a small proportion of *nad2* 280 C was changed to U (Fig. S6). Compared to the WT *nad2* editing status, no editing sites were affected in *mid*1 mutants (Fig. S6). Taken together, *mid1* is severely impaired in splicing of *nad2* intron 1.

To investigate whether mitochondrial respiration complexes were affected, blue native gel electrophoresis (BN-PAGE) was performed. Complex I of mitochondrial respiration chain is a large protein complex consisting of more than 40 subunits encoded by both nuclear and mitochondrial genomes, among which mitochondrial genome-derived *nad2* subunit localizes at the membrane arm of Complex I^77^. As the above results indicating that the splicing of *nad2* first intron was deficient in *mid1* mutants, we next investigated whether this failure of splicing affects Complex I. Meanwhile, a previously reported Complex I deficient mutant *mtsf1* (Salk_086724) was used as a control^78^. Mitochondria were isolated from 4-week-old wild-type, *mtsf1,mid1* mutants and the complemented plants, followed by BN-PAGE resolution and Coomassie staining, to detect the integrity and activity of Complex I. Distinct complexes can be distinguished according to the analogous separation results done before^79^. Similar to *mtsf1*, there was a remarkable reduction of Complex I abundance in *mid1* mutants compared with the wild-type and the complemented plants, indicating that the integrity of Complex I was impaired in *mid1* mutants (Fig. 5C). Consistently, further enzymatic detection showed that the activity of Complex I decreases dramatically in *mid1* mutants (Fig. 5D). These results confirmed that the *nad2* intron 1 splicing deficiency in *mid1* further impairs the Complex I function.

To further investigate whether the mitochondrial function deficiency had effect on their morphology, we performed the transmission electron microscopy to check the ultrastructure of mitochondria in the fifth leaf of 4-week-old wild-type and *mid1-1* plant. In the mesophyll cells, there were two striking morphological differences between wild-type and *mid1-1*. First, nearly 70% of mitochondria (n = 140) has altered cristae structural organization in *mid1-1*. Cristae was abundant and arranged regularly in the wild-type (Fig. 5E), but less abundant and disordered in *mid1-1* (Fig. 5F). Second, mitochondrial size was swollen in *mid1-1*, about 1.3 times that of the wild-type (Fig. 5G). These findings indicate that loss-function of *MID1* results in compromised mitochondrial ultrastructure.

As reported above, mitochondrial respiration Complex I was disrupted in *mid1* mutants due to the failure of *nad2* intron 1 splicing. The electron transport along the mitochondrial respiration complexes is the prerequisite for the establishment of proton gradient cross the inner membrane, which further ensures the successful generation of ATP^80,81^. Therefore, we reasoned that the impaired Complex I activity may influence the mitochondrial energy metabolism. To test this hypothesis, we measured the ATP content in the leaf of wild-type and *mid1-1*. The result showed that ATP content dramatically decreased in *mid1-1* compared to the wild-type (Fig. 5H), consistent with the previous results that functional deficiency in Complex I impairs ATP production^82,83^. Reactive oxygen species (ROS) are inevitable byproducts originally generated by excess electron transferred to O_2_, which ultimately give rise to H_2_O_2_^84,85^. Previous studies have revealed that disruption of Complex I could bring about the accumulation of H_2_O_2_^54,86^. To access whether *mid1-1* produces more H_2_O_2_, we measured the H_2_O_2_ content through 3′, 3′- diaminobenzidine (DAB) staining, which clearly showed that more H_2_O_2_ accumulated in *mid1-1*_,_ especially in the vascular tissue (Fig. S7A,B), Consistently, subsequent quantitative assay indicates that H_2_O_2_ in *mid1-1* was 1.5 times more than that of the wild-type (Fig. S7C). Together, these results suggest that the malfunctional mitochondrion caused by *MID1* mutation further lead to production of less ATP and more H_2_O_2_ than the wild-type.

### MID1 is associated with other *trans*-factors involved in *nad2* intron 1 splicing

**Figure 6:**
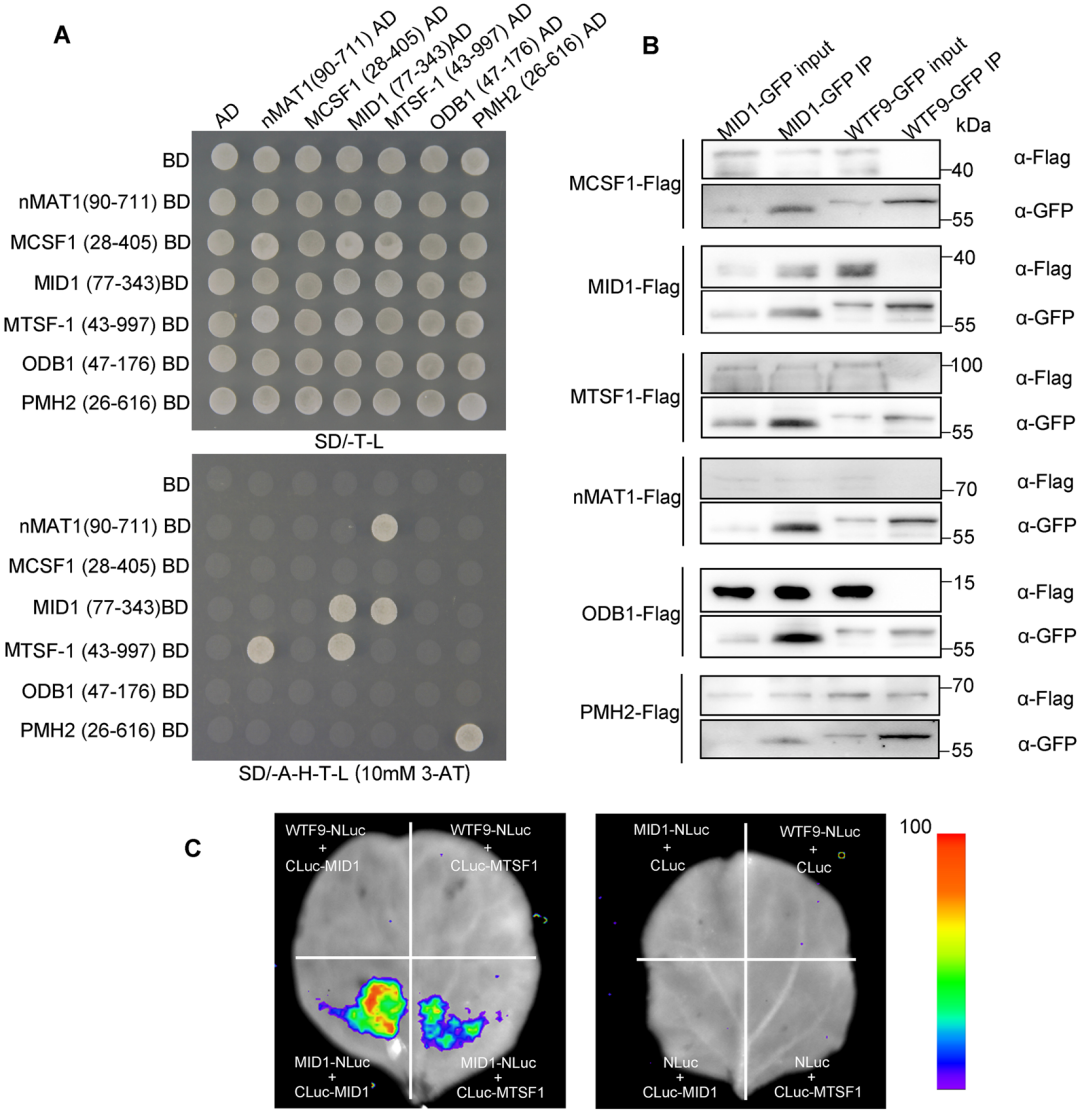
MID1 is associated with other *trans*-factors involved in *nad2* intron 1 splicing. (**A**) Y2H analysis of interaction relationship between *trans*-factors involved in *nad2* intron 1 splicing. The upper panel displays the yeast that can grow on the SD/-Trp-Leu (SD/-T-L) dropout media. The lower panel displays the yeast growing on the SD/-Ade-His-Trp-Leu (SD/-A-H-T-L) dropout media, which indicates the physical interaction of two *trans*-factors. 10 mM 3-amino-1, 2, 4-triazole (3-AT) is added in the media to inhibit the autoactivation of MTSF1(43–997aa) BD + AD and BD + ODB1(47–176aa) + AD. (**B**) Co-IP analysis of *trans*-factors association in *Arabidopsis* protoplast. About 70 μg total proteins were loaded in the input lanes and one sixth volume of GFP beads were loaded in the IP lanes. Protein mark is on right side of immunoblot. (**C**) Luciferase complementation assay of interaction between MID1, MTSF1 and itself. The pseudocolor bar indicates the relative luminescence intensity in the image.

Group II intron, first found in fungi and plant organelle genomes, is a self-splicing ribozyme that catalyzes auto-excision *in vitro*, but it requires the assistance of *trans*-factors to fulfill this process *in vivo*^87–89^. As previously reported, there are five *trans*-factors also involved in the splicing of the *nad2* intron 1 in *Arabidopsis*, including MITOCHONDRIAL CAF-LIKE SPLICING FACTOR 1 (MCSF1), MTSF1, nMAT1, ORGANELLAR DNA-BINDING PROTEIN 1 (ODB1) and PUTATIVE MITOCHONDRIAL RNA HELICASE 2 (PMH2)^55,59,78,90,91^. We reasoned if MID1 may work together with these *trans*-factors to promote *nad2* intron 1 splicing. In order to explore this possibility, we first examined their interaction relationship by yeast two-hybrid (Y2H) assay. As shown in Fig. 6A, MID1 could interact with MTSF1 and itself in yeast. Meanwhile, we also detected that nMAT1 can interact with MTSF1, and PMH2 itself can form homodimer. Next, we performed Coimmunoprecipitation (Co-IP) assays in *Arabidopsis* protoplasts to investigate whether they are in the same complex to control *nad2* intron1 splicing. MID1-GFP is transiently co-expressed with each Flag-tagged *trans*-factor in the *Arabidopsis* protoplasts. WHAT’S THIS FACTOR 9(WTF9), a mitochondria-localized protein involved in *ccmFc* and *rpl2* introns splicing^92^, was fused with GFP to act as the negative control. As shown in Fig. 6B, all the *trans*-factors tagged with Flag could be successfully pulled down by the MID1-GFP, while WTF9-GFP failed to pull down the target proteins including MCSF1, MID1, MTSF1, nMAT1 and ODB1. WTF9 can pull down PMH2 because they are involved in splicing of *rpl*2 intron 1^93^, which indicates that they work together to promote intron splicing. Together, WTF9 IP control results point that MID1 interact specifically with other *trans*-factors, however we can’t exclude the possibility that RNA may facilitate their interactions. In order to further confirm the interaction between MID1, MTSF1 and itself, we performed firefly luciferase complementation imaging assay in tobacco leaves^94^, in which WTF9 acted as the negative control. As shown in Fig. 6C, while no LUC activity was found in the combination of WTF9-NLuc/CLuc-MID1, WTF9-NLuc/CLuc-MTSF1, both the combination of MID1-NLuc/CLuc-MTSF1 and MID1-NLuc/CLuc-MID1 showed Luc activity, indicating that MID1 can interact with MTSF1 and itself in tobacco. Although MID1 has association with all the other *trans*-factors, only few of them have direct physical interactions, which can be ascribed to additional uncharacterized *trans*-factor or RNA that can mediate the physical interaction between these *trans*-factors. When we finished the interaction assays, another PLS-type PPR protein SLO4 was reported to influence *nad2* intron 1 splicing^50^, it is worthy to test its interaction relationship with other *trans*-factors.

MTSF1 is a mitochondria-localized large PPR protein with 19 PPR motifs which positively regulate the stability of *nad4* mRNA and *nad2* intron 1 splicing^78^. Regarding that MID1 can interact with MTSF1, it is reasonable to infer that MID1 may also be involved in *nad4* mRNA stability. No *nad4* mRNA was detected in *mtsf1* mutants in the previous report. However, the abundance of *nad4* mRNA in *mid1* mutants was similar to that of wild-type (Fig. 5B). Thus, our results suggest that MID1 is not involved in *nad4* mRNA stability and the interaction between MID1 and MTSF1 mainly functions in the splicing of *nad2* intron 1.

### The N-Terminal (77–135aa) and the PPR motifs are important for *nad2* intron 1 splicing and plant development

In order to identify the domain responsible for MID1 dimerization, a variety of truncation and fusions lack of different domains of MID1 were constructed and tested in yeast. The MID-BD variants with the first PPR motif (136–170aa), which include MID1(77–170aa)BD, MID1(77–205aa)BD, MID1(77–240aa)BD, MID1(77–276aa)BD, MID1(77–311aa)BD, MID1(77–343aa)BD and MID1(136–170aa) BD were able to interact with MID1(77–343)AD, whereas MID1 variant only containing the N-terminus, MID1(77–135)BD, failed to interact with MID1(77–343)AD. Furthermore, first PPR motif itself, MID1(136–170) BD can interact with MID1 variants that include MID1(77–170aa)AD, MID1(77–205aa)AD, MID1(77–240aa)AD, MID1(77–276aa)AD, MID1(77–311aa)AD and MID1(77–343aa)AD, while MID1(136–170)BD can’t interact with MID1(136–170)AD (Fig. S9a). These results showed that the first PPR domain mediates the dimerization and the N-terminus facilitate the homodimer formation.

**Figure 7:**
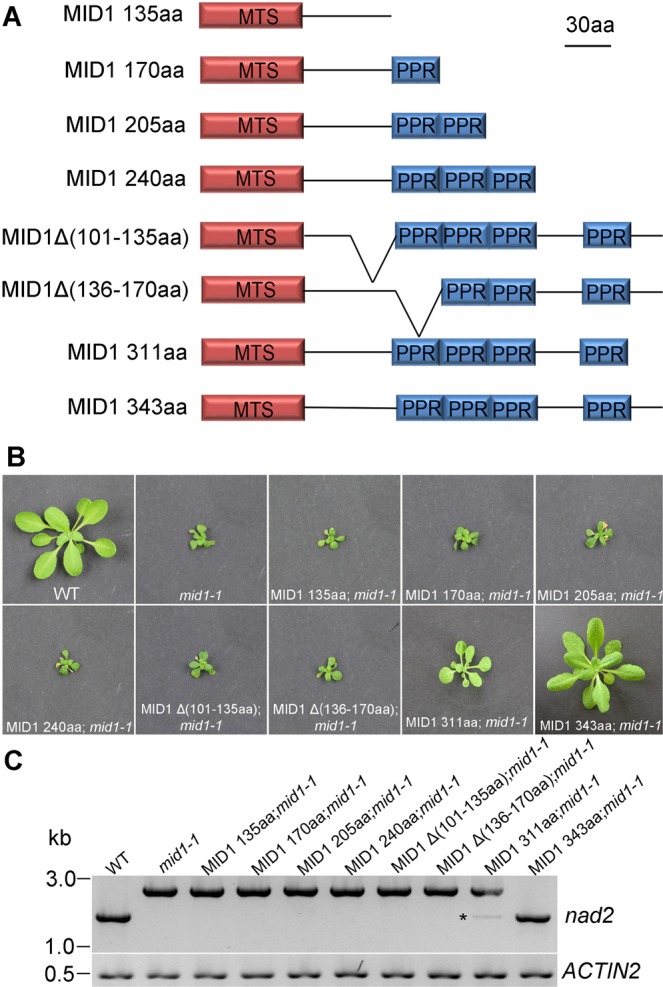
Functional analysis of each domain of *MID1*. (**A**) Schematic diagram of *MID1* variants. (**B**) The phenotypes of *mid1-1* complemented by *MID1* variants. Bar = 1 cm. (**C**) RT-PCR analysis of splicing efficiency of *nad2* intron 1 in *mid1-1* complemented by *MID1* variants. *ACTIN2* is used as loading control.

We further characterized the role of each domain in MID1 in RNA splicing and plant development. To explore this, a variety of MID1 variants were constructed, which include MID1(135aa), MID1(170aa), MID1(205aa), MID1(240aa), MID1Δ(101–135aa), MID1Δ(136–170aa), MID1 (311aa) and full-length MID1(343aa) (Fig. 7A). These variants, driven by 35S promoter and fused with GFP at its C-terminus, were subsequently transformed to the *mid1-1*/+ plants for genetic complementation. As shown in Fig. 7B, neither MID1 variants lack the N-terminus (101–135aa) nor were the ones lacking PPR motif able to rescue *mid1-1* growth phenotype, indicating that the N-terminus and PPR motifs are essential for MID1 function in plant development. On the other hand, the full-length of MID1 could completely rescue *mid1-1* and the MID1 variant without the C-terminus (312–343aa) partially rescue *mid1-1*, indicating that the C-terminus (312–343aa) plays a less important role during plant development. Consistently, the splicing efficiency of *nad2* intron 1 correlated with the complementation level (Fig. 7C). While the MID1 variant without the C-terminus (312–343aa) could partially restore the splicing efficiency of *nad2* intron 1, there was no alternation of splicing efficiency in the transgenic plant with the other MID1 variants lacking the N-terminus or a PPR motif, suggesting that the N-terminus and the PPR motifs are necessary for *nad2* intron 1 splicing.

## Discussion

Here, we isolated an embryo-defective mutant *mid1* which displays growth defects during the whole plant life cycle. *MID1* encodes a mitochondria-localized short P-type PPR protein essential for the correct splicing of the first intron of *nad2*, mutation of which disrupts the assembly of mitochondrial respiration Complex I and the ultrastructure of mitochondria. Furthermore, MID1 is found to be associated with other *trans*-factors, among which MID1 can interact with itself and MTSF1 to form homodimer and heterodimer, respectively. The genetic complementation further confirms that the N-terminus and each PPR motif are required for *nad2* intron 1 splicing and plant development. In summary, MID1 is a novel short P-type PPR protein required for *nad2* intron 1 splicing, mitochondrial biogenesis and plant development.

### MID1 plays roles in RNA splicing likely in a dimeric fashion

In contrast to most PPR proteins involved in RNA splicing which are usually long in protein length and harbor more than ten PPR motifs, MID1 is a short P-type PPR protein with only four canonical PPR motifs, similar to another well characterized short PPR protein THA8^29^. THA8 is a chloroplasts-localized PPR protein required for the efficient splicing of *ycf3-2* and tRNA introns. *In vitro* biochemical experiments have validated that THA8 orthologues can bind *ycf3*-*2* intron through multiple purine-rich sequences distributing in intron^30^. More importantly, the crystal structure analysis of THA8 and THA8-RNA reveals that RNA targets can induce THA8 to form dimer and oligomer which will fold the linearized intron RNA into a condensed tertiary structure that can be easily bound by other splicing factors^21,30^. Similarly, in our yeast assays, we found that the MID1 can form dimer, in which the first PPR motif of MID1 directly mediate the dimerization and the N-terminus (77–135aa) can facilitate this formation process. It seems that some PPR proteins fulfill their distinctive functions in a dimer manner. How the N-terminus exert effects on the dimerization is still unknown. Experimental identification of RNA targets of MID1 and resolution of MID1/MID1-RNA crystal structure in the further will elucidate the action mode of MID1 in detail. PPR10 is one of the best characterized PPR proteins, but its action mode is still uncertain. The dispute about whether PPR10 works as dimer or monomer may originate from that they use different strategies to study PPR10^24,35^. It is worthy to figure out its real physiological state *in vivo*. In addition, it seems that there is no common manner adopted by all PPR proteins to perform function. While some PPR proteins work as dimer^28,30^, some others work as monomer^31,32^.

### MID1 indirectly affects cell expansion and proliferation

*mid1* displays growth retardation and dwarfism phenotypes during the whole plant life cycle, which can be ascribed to its impaired cell expansion and proliferation. The limited production of ATP caused by abnormal mitochondrial biogenesis may be in part responsible for these cellular defects. In mammals, AMP-ACTIVATED PROTEIN KINASE (AMPK) is widely considered to be the cellular energy sensor which can be activated by nutrient stress to maintain energy homeostasis^95^. One of these cellular effects exerted by AMPK in energy-deficient cell is to arrest cell cycle through phosphorylation p53 and transcriptional activation of cyclin-dependent kinase inhibitor 1A (CDKN1A)^96^. SUCROSE NON-FERMENTING 1 PROTEIN (SNF1)-RELATED PROTEIN KINASE 1 (SNRK1), the atypical AMPK orthologue in *Arabidopsis*, has been shown to participate in ABA and sugar signaling^97,98^, whether it is also involved in the regulation of cellular stress response induced by dysfunctional mitochondrion remains elusive. Alternatively, there are evidences that many other signaling pathways such as ABA, Auxin and GA signaling may act downstream of compromised mitochondrion to convey the energy shortage information^54,86,99^. Thus, it seems that the abnormal hormone signaling may commonly underlie the cellular deficiency of PPR mutants, which may be further responsible for their mutant phenotypes. Besides, great advances have been made in understanding the plant mitochondrial retrograde signaling in the past decade. For example, many important transcription factors downstream of mitochondrial retrograde signaling have been identified through forward and reverse genetic study, such as ABIA INSENSITIVE 4 (ABI4), WRKY40, WRKY63, NO APICAL MERISTEM/ARABIDOPSIS TRANSCRIPTION ACTIVATION FACTOR/CUP-SHAPED COTYLEDON (ANAC013), ANAC017 and MYB DOMAIN PROTEIN 29, which positively or negatively regulate nuclear genes expression upon mitochondrial stress^100–104^. It will be interesting to genetically evaluate whether they contribute to the mutant phenotypes caused by MID1 disruption.

In conclusion, we characterized a mitochondria-localized PPR protein MID1 which is required for the mitochondrial RNA splicing and *Arabidopsis* development. The association of *trans*-factors involved in *nad2* intron 1 splicing will lay the foundation for further structural resolution of the mitochondrial RNA splicing apparatus by means of single-particle cryogenic electron microscopy which has successfully resolve the three-dimensional structure of yeast spliceosome at near atom level^105^. Finally, the mitochondrial retrograde signaling may contribute a lot to the abnormal morphology of *mid1*, thus *mid1* is a candidate mutant that is instrumental to discover the novel components involved in the mitochondrial retrograde signaling in a genetic manner.

### Plant materials and growth conditions

Both *Arabidopsis thaliana* ecotype Landsberg *erecta* (L*er*) and Columbia-0 (Col-0) were used as wild-type here. *mid1-1*(*Ds* insertion line) was in L*er* background while the cell proliferation reporter line *CYCB1;1-GUS*, *mtsf1* (SALK_086724) and *mid1-2* (CRISPR/Cas9-derived mutant) was in Col-0 background. *CYCB1;1-GUS* reporter used in this study was as previously described^64^. SALK_086724 was obtained from ABRC stock center. *mid1-2* was a CRISPR/Cas9-derived mutant. Seeds sterilization and genetic transformation were performed as previously reported^53,54^. The plants were grown in the air-conditioned house (22 °C) under a 12 h light/12 dark cycle.

### Phenotypic analysis

To investigate the embryogenesis phenotype, siliques of *mid1*/+ plants were dissected with hypodermic needles and cleared in Herr’s solution^106^. The cleared ovules were subsequently observed with Zeiss Axioskop II microscope equipped with differential interference contrast optics. Measurement of cell size of leaf and root was performed as reported^57^.

### Molecular cloning and genetic complementation

Thermal asymmetric interlaced PCR was performed to identify *MID1* that is disrupted by *Ds* insertion. To complement the *mid1-1* mutant, the 35S promoter and the NOS terminator sequences were cloned into *pCAMBIA1300* to generate *pCAM1300-35SPro-NOS*. Then, we amplify the *GFP*,*GUS* and 3 × *Flag* sequence and inserted them into *pCAM1*3*00-35SPro-NOS* to produce *pCAM1300-35SPro:GFP-NOS*, *pCAM1300-35SPro:GUS-NOS* and *pCAM1300-35SPro:3* × *Flag-NOS*, respectively. To produce *MIDPro:MID1-GUS* and *MIDPro:MID1*-3 × Flag, 35S promoter was cut from *pCAM1300-35SPro:GFP-NOS* and *pCAM1300-35SPro: GUS-NOS* by restriction enzymes digestion. The *MID1* promoter and *MID1* coding sequence were amplified with gene specific primers in Supplemental Table and simultaneously inserted into *pCAM1300-GUS-NOS* and *pCAM1300-3* × *Flag-NOS* through in-fusion clone (638933, Takara). To produce the *35SPro:MID1* variants-*GFP*, the full-length *MID1* and the truncated *MID1* coding sequence were amplified with specific primers in Supplemental Table and were introduced into *pCAM1300-35SPro:GFP-NOS*. All the constructs were transformed into *Agrobacterium tumefaciens* strain GV3101, which were then introduced into *mid1-1*/+ plant through *A. tumefaciens*-mediated floral dip transformation^107^.

### Microscopy

For GUS assay, various organs or the whole seedling of *MID1Pro:MID1-GUS* complementation lines were immersed in GUS staining solution as reported previously^54^. For confocal microscopy, 6-day-old seedlings of *35SPro:MID1-GFP* complementation lines were stained with 500 nM MitoTracker^®^ Red CMXRos (M7512, Invitrogen/Molecular Probes) for 5 min, followed by washing three times using the half-strength MS liquid medium. The stained cotyledons were observed with a 488 nm and 543 nm laser of a Zeiss CLSM laser scanning microscope. Transmission electron microcopy was performed as previously reported^108^.

### Quantitative RT-PCR (qRT-PCR)

Total RNA from various organs were extracted using the plant RNeasy mini-kit (74104, Qiagen). First-strand cDNA was then synthesized from 1–5 μg of RNA using the superscript III reverse transcriptase (18080093, Thermo Fisher) with the oligo-dT primer according to the manufacturer’s instructions. For qRT-PCR analysis, the primers used to amplify *MID1* were designed by Beacon Designer 8 software while primers for analysis of splicing ratio of mitochondrial RNA transcripts was performed as described previously^39^. qRT-PCR was performed with SYBR Green SuperReal PreMix Plus (FP205, TIANGEN) on the Bio-Rad C1000 Thermal Cycler.

### Mitochondrion and chloroplast isolation

Mitochondria isolation was performed according to the previously reported protocol with minor modification^109^. A total of 20 g soil grown *Arabidopsis* plants were ground in pre-cold grinding solution [300 mM sucrose, 10 mM KH_2_PO_4_ (PH7.5), 25 mM Na_4_P_2_O_7_, 1 mM EDTA, 1% (w/v) bovine serum albumin (BSA), 1% [w/v] PVP40, 10 mM cysteine, 1 mM DTT and 1x proteinase inhibitor cocktail]. Tissue homogenate was further broken by Teflon homogenizer at 100 rpm–150 rpm. The resulting cell debris were filtered through two layers of miracloth (473855, Millipore) and separated at 3000 g for 5 min at 4 °C to remove the nucleus. The supernatants were further subject to centrifugation at 15000 g for 30 min to obtain the crude organelle pellets which were subsequently recovered by washing buffer (300 mM sucrose, 10 mM MOPS, 1 mM EGTA). Repeating the differential centrifugation described above. The final suspended homogenate was carefully layered on the discontinuous Percoll (P1644, Sigma) density gradients comprised of 18%, 26%, 50% Percoll (1:5:1, v/v). After centrifugation at 40000 g for 45 min, mitochondria were collected from the interface of 26%/50% and washed three times using washing buffer to eliminate the Percoll. The purified mitochondrial protein concentration was analyzed by Branford protocol and can be directly frozen at −80 °C for the future use.

The isolation of chloroplast was performed according to the previously described protocol with some modifications^110^. *Arabidopsis* leaves were homogenized in isolation buffer (50 mM HEPES-KOH, pH 7.8, 330 mM sorbitol, 1 mM EDTA, 1 mM EGTA, 10 mM Na_2_CO_3_, 0.1% (w/v) BSA and 50 mM ascorbate [freshly added]) with 1× protease inhibitor cocktail. The homogenate was filtered through two layers of Miracloth (475855, Millipore) and then centrifuged for 5 mins at 2000 g. The collected crude chloroplasts were resuspended in 500 μL separation buffer (50 mM HEPES-KOH, pH 7.8, and 330 mM sorbitol) and laid on the 40%/70% Percoll gradient. After centrifugation for 5 mins at 1500 g, the intact chloroplasts were collected between 40% and 70% Percoll gradient and washed twice with the separation buffer. The purified chloroplasts can be stored at −80 °C for further use.

### Blue native page and immunodetection

About 100 μg extracted mitochondria were solubilized in cold NativePAGE Sample Buffer (BN2003, ThermoFisher) containing 1% dodecilmaltoside (DDM) according to the manufacture’s instruction. The mitochondrial protein complexes were separated using the 4–16% NativePAGE Bis-Tris Gel (BN1002BOX, Thermo Fisher) which was further used for Coomassie brilliant blue staining, the Complex I activity assay or transferred to PVDF membrane for immunodetection. Coomassie brilliant blue staining and immunodetection were performed according to the manufacturer’s instruction. In-gel detection of Complex I activity was performed according to the protocol described before^111^.

### Y2H assay

The coding sequence of *MCSF1*, *MID1* variants, *MTSF1*, *nMAT1*, *ODB1* and *PMH2*, without the N terminal mitochondrial targeting signals, were cloned to the bait vector *pGBKT7* and prey vector *pGADT7* (Clontech), respectively. The primers are listed in Supplemental Table. Yeast transformation was carried out according to the protocol described in the manual’s instruction (Clontech). In the detection of some protein interaction pairs, 3-AT was added to prevent their auto-activations.

### Protoplast transformation and Co-IP

The full-length *MID1* and *WTF9* coding sequences were cloned into *pBluecript II SK*(+)*35Pro:GFP* and the full length *MCSF1*, *MID1*, *MTSF1*, *nMAT1*, *ODB1* and *PMH2*, were cloned into *pBluecript II SK*(+)*35SPro:3* × *Flag*^112^. Tags were localized at C-terminal of genes. *Arabidopsis* protoplast was prepared from 3~4 weeks old plants growing in the12 h light/12 h dark condition. The subsequent plasmids transformation was performed according to the method described previously^113^. For Co-IP assay, total protein was extracted with extraction buffer (50 mM Tris-Cl pH 7.5, 150 mM NaCl, 1 mM EDTA, 0.5% NP40, 1 mM DTT, 1 × proteinase inhibitor cocktail). The pre-equilibrated GFP Trap beads (gta-10, Chromotek) incubates with protein extracts overnight at 4 °C. The GFP Trap beads were washed five times with washing buffer (50 mM Tris-Cl pH 7.5, 150 mM NaCl, 1 mM EDTA, 1% NP40, 1 mM DTT, 1 × proteinase inhibitor cocktail). Resuspend the GFP beads with a small volume of washing buffer, followed by boiling them for 5 mins. The eluted protein was ready for SDS page separation and immune-blot assay using the anti-GFP (M20004M, Abmart) and anti-Flag (F1804, Sigma).

### Firefly luciferase complementation imaging assay

The corresponding sequences of *MID1* and *WTF9* were inserted into *pCAMBIA-NLuc* to produce *MID1-NLuc* and *WTF9-NLuc*. The corresponding sequences of *MID1* and *MTSF1* were inserted into *pCAMBIA-CLuc* to generate *CLuc-MID1* and *CLuc-MTSF1*. Agrobacterium-mediated infiltration of *N. benthamiana* leaves was performed as described^94^. Infiltrated leaves were incubated at 22 °C for 72 h before CCD imaging.

### ATP and H_2_O_2_ assay

ATP content was measured as described earlier^83^. The detection of H_2_O_2_ was performed as reported previously^114^. Leaves of wild-type and *mid1-1* were subject to quantitative measurement of H_2_O_2_ using the Amplex^®^ Red Hydrogen Peroxide/Peroxidase Assay Kit (A22188, Invitrogen). The assay was performed according to the manufacturer’s instruction.

### Accession numbers

Sequence data can be found in the *Arabidopsis* Genome Initiative database or the GenBank/EMBL data library under the following accession numbers: *ACTIN2* (*At3g18780*), *MCSF1* (*At4g31010*), *MTSF1* (*At1g06710*), *MID1* (*At1g06270*), *nMAT1* (*At1g30010*), *ODB1* (*At1g71310*), *PMH2* (*At3g22330*) and *WTF9* (*At2g39120*).

## Supplementary information

supplemental data.
