# Supplementary material for: Pentatricopeptide repeat protein MID1 modulates nad2 intron 1 splicing and Arabidopsis development
Source: Sci Rep. 2020 Feb 6;10:2008. doi: 10.1038/s41598-020-58495-5 (PMC7005036; doi:10.1038/s41598-020-58495-5)
Supplement: Supplementary file 1 — supplemental data. [file 41598_2020_58495_MOESM1_ESM.pdf]

Pentatricopeptide repeat protein MID1 modulates *nad2* intron 1 splicing and *Arabidopsis* development

Peng Zhao[1,2], Fang Wang[1,2], Na Li[1], Dong-Qiao Shi[1] & Wei-Cai Yang[1,*]

[1]State Key Laboratory of Molecular and Developmental Biology, Institute of Genetics and Developmental Biology, Chinese Academy of Sciences, Beijing 100101, and University of Chinese Academy of Sciences, Yuquan Road, Beijing 10049, China

[2] These authors contributed equally to the article.

[*]Corresponding author: wcyang@genetics.ac.cn.

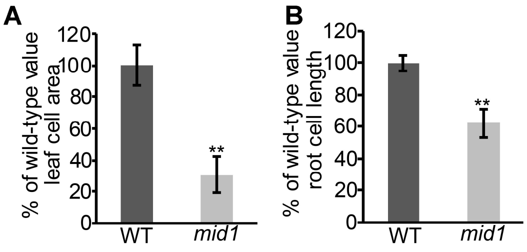

Supplemental Figure 1 The cell expansion deficiency of *mid1*.

(A) The fifth leaf cell area of wild-type and *mid1*. Data are the mean ± SE, n=100. Student's t tests, **P<0.01. **(B)** The cell length of wild-type and *mid1* in the root maturation region. Data are the mean ± SE; n=50. Student's t tests, **P<0.01.

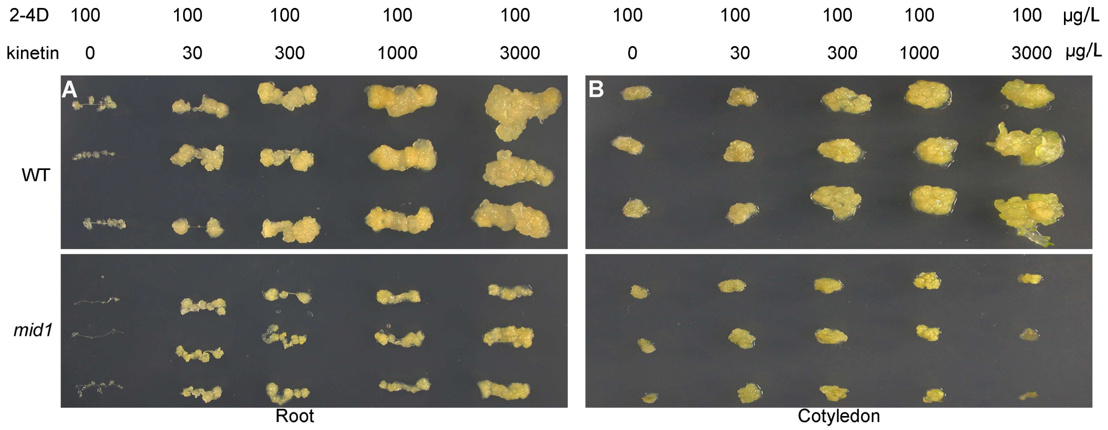

Supplemental Figure 2 Callus Induction on the solid MS medium under darkness

(A) Callus growth from primary root segments of wild-type (upper panel) and *mid1* (lower panel) after 3-week-induction. **(B)** Callus growth from cotyledon segments of wild-type (upper panel) and *mid1* (lower panel) after 3-week-induction.

```
mid1-2   M   A   I   R   L   T   H   L   R   K   P   L   A   Y   S   R   S   F
         GAT GTC TGC ATT CCG TTT TTC CGG TCA ATC TCG TCC TTT GAA GCG GTT GAA AAG 108bp
WT       D   V   C   I   P   F   F   R   S   I   S   S   F   E   A   V   E   K
         GAT GTC TGC ATT CCG TTT TTC CGG TCA ATC TCG TCC TTT GAA GCG GTT GAA AAA
mid1-2   D   V   C   I   P   F   F   R   S   I   S   S   F   E   A   V   E   K
WT       GCG ATA AAA TGT GCT GTT GAA ACA AAG GAG TAT CTT AGA ATC --------
         A   I   K   C   A   V   E   T   K   E   Y   L   R   I
mid1-2   GGC GAT AAA ATG TGC TGT TGA AAC AAA GGA GTA TCT TAG AAT
         G   D   K   M   C   C   *
```

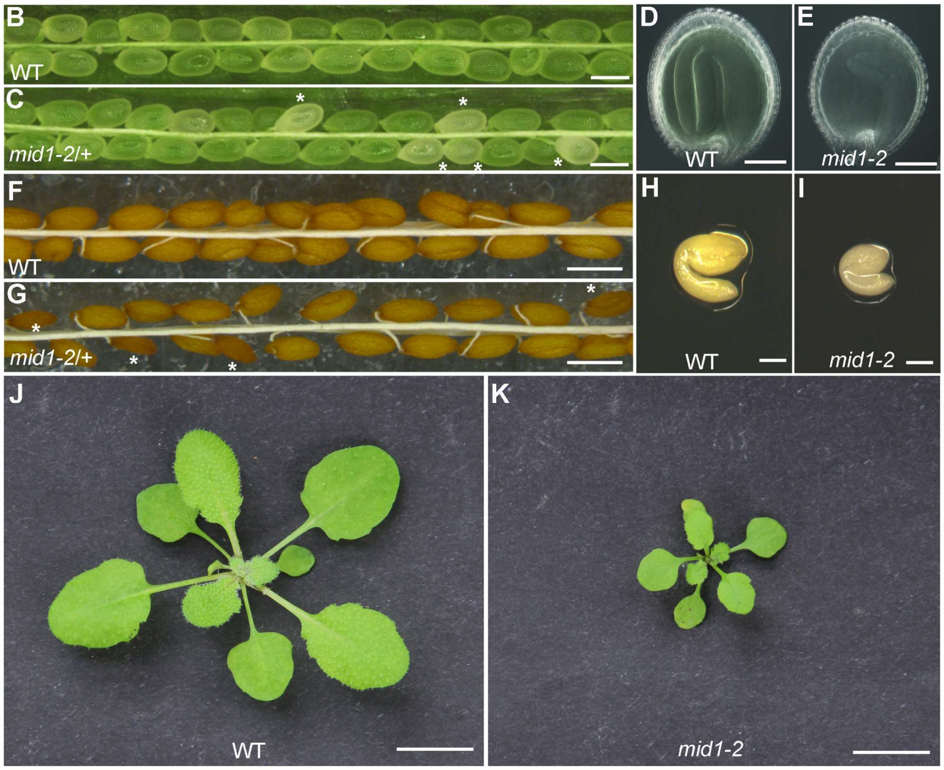

Supplemental Figure 3 Phenotypes of *mid1-2*

(A) CRISPR–Cas9-induced mutation in *mid1-2*. The rectangle marks the target sequence. The red A indicates the nucleoside inserted in *mid1-2* and a premature stop codon was created after six amino acid. The truncated MID1 in *mid1-2* is 42 amino acid in length. **(B)-(C)** Seed set of wild-type and *mid1-2*/+ siliques at 9 DPP. Wild-type shows a full seed set while approximately one-quarter of ovules in the *mid1-2*/+ silique are abnormal. Asterisks indicate the *mid1-2* ovules. **(D)-(E)** Whole-mounted ovules from silique of *mid1-2*/+ plant in **(C)**. **(F)-(G)** Seeds in the mature silique of wild-type and *mid1-2*/+. Compared to the wild-type, about one-quarter of the seeds in *mid1-2*/+ display shrunken morphology. Asterisks indicate the *mid1* seeds. **(H)-(I)** Embryos dissected from *mid1-2*/+ plant in **(G). (J)** 4-week-old wild-type plant at short day condition. **(K)** 4-week-old *mid1-2* plant at short day condition.

(B), (C), (F), (G), Bars=200μm. **(D), (E), (H), (I),** Bars=50μm. **(J), (K),** Bars=1cm**.**

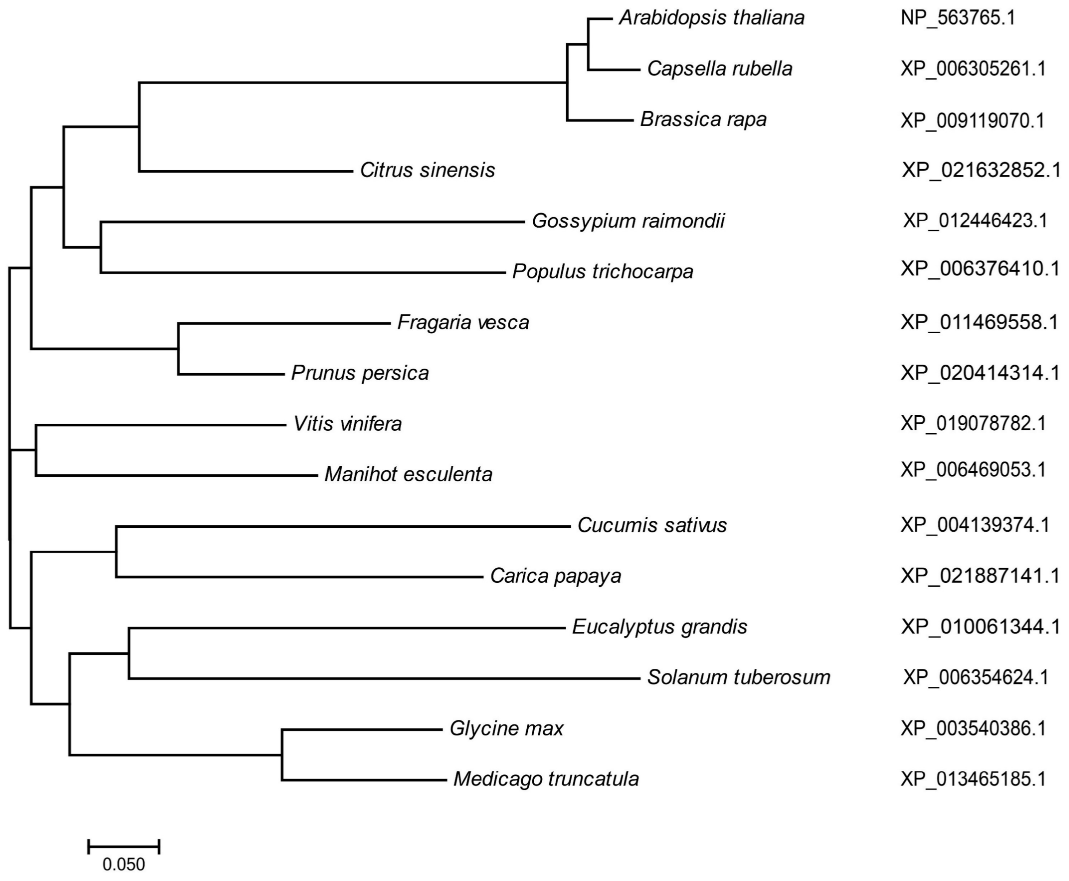

Supplemental Figure 4 Phylogenetic analysis of MID1 orthologues

The MID1 orthologues protein sequences of sixteen species are derived from NCBI (https://www.ncbi.nlm.nih.gov/). They are aligned to generate the maxium-likehood phylogenetic tree using MEGA6. The gene locus or GenBank/EMBL accession numbers of these orthologues are listed in the right panel. Branch length represents substitutions per site.

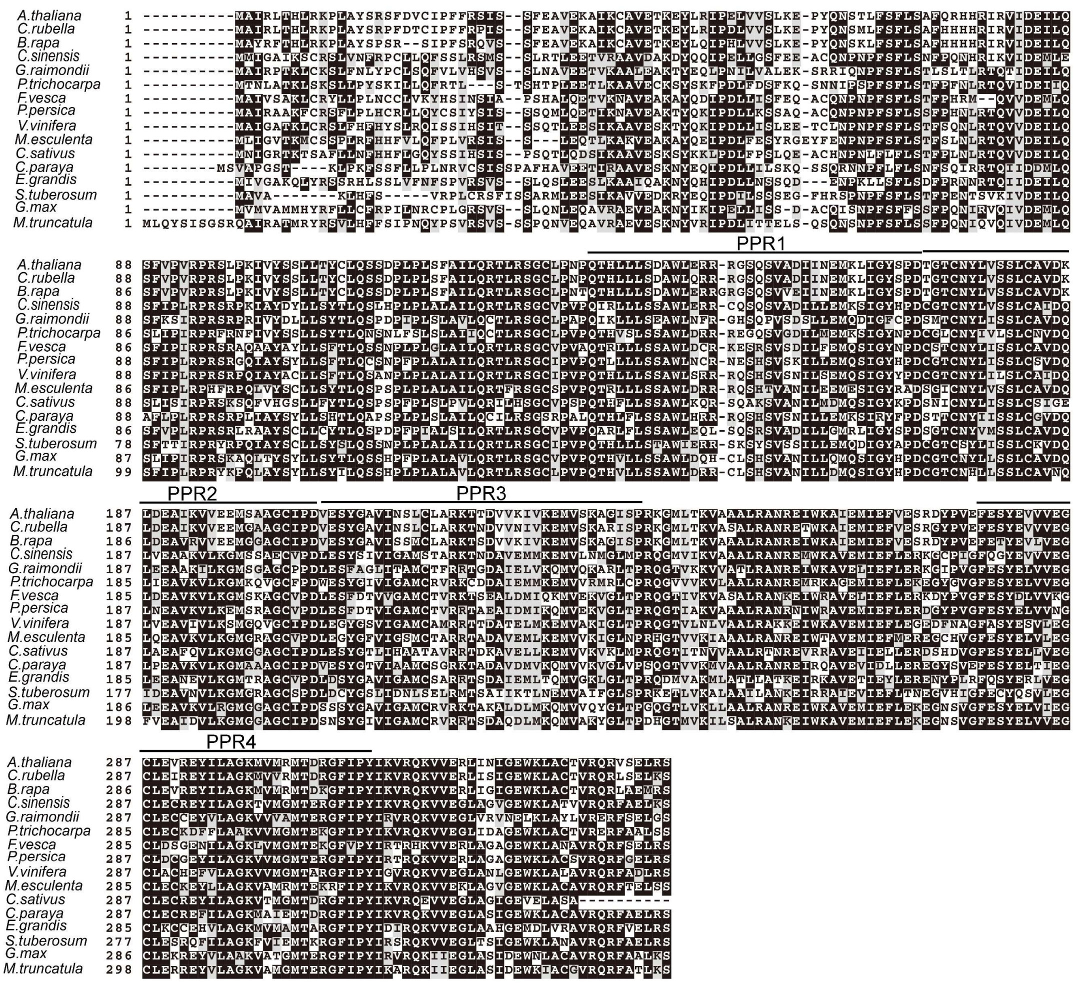

Supplemental Figure 5 Alignment of MID1 orthologues.

The conserved PPR domains are indicated by black line on the top.

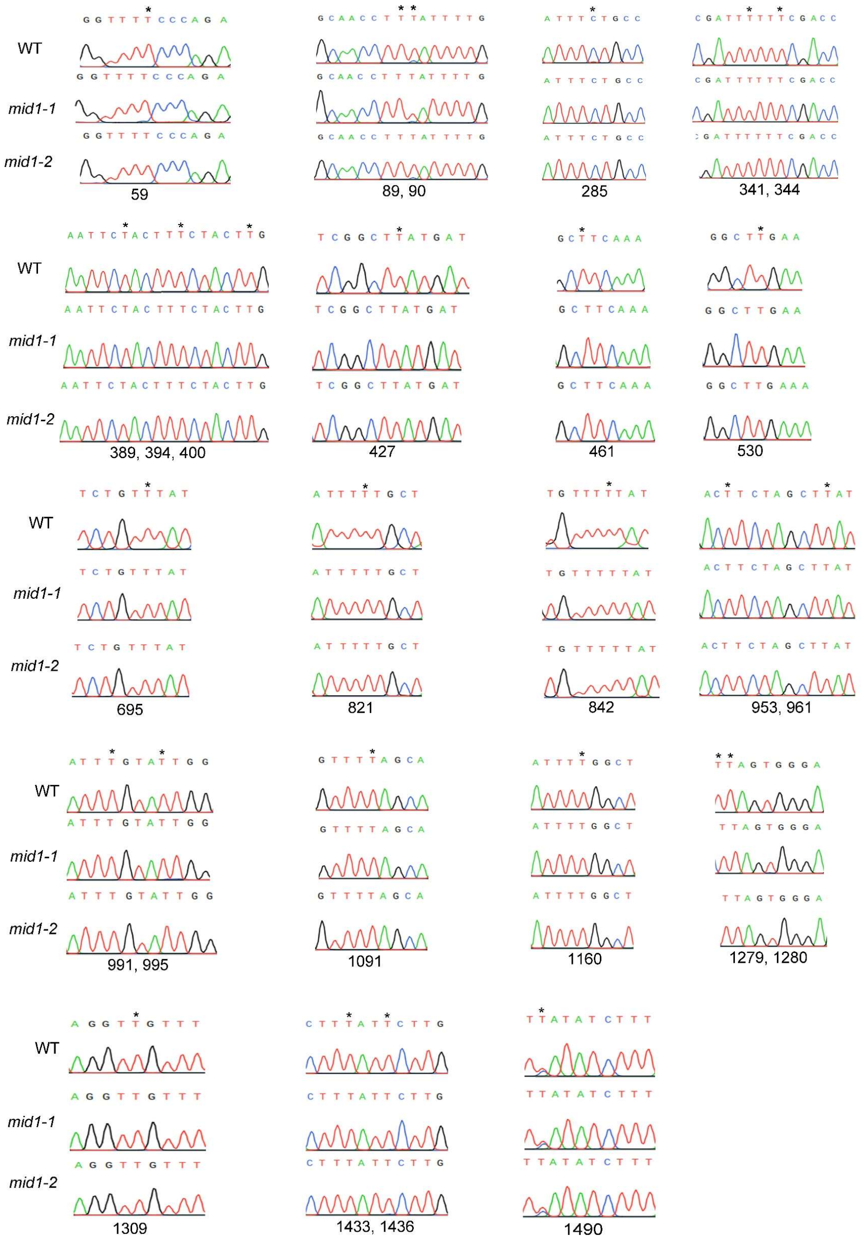

Supplemental Figure 6 *nad2* editing status in wild-type and *mid1* mutants.

The asterisks indicate the editing sites. The number under the sequence map marks the position of each editing site.

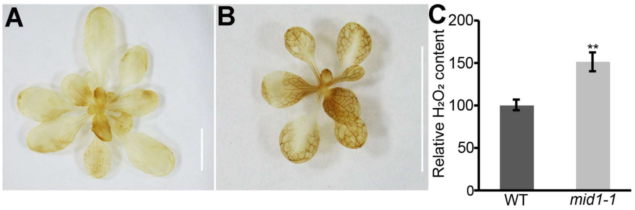

Supplemental Figure 7 Measurement of H_2O_2 content in *mid1*

DAB staining detection of H_2O_2 in 4-week-old wild-type **(A)** and *mid1-1*plant **(B)**. Bars=1cm.

(**C**) Quantitative assay of H_2O_2 content in **(A)** and **(B)**, Student's t tests, **P<0.01.

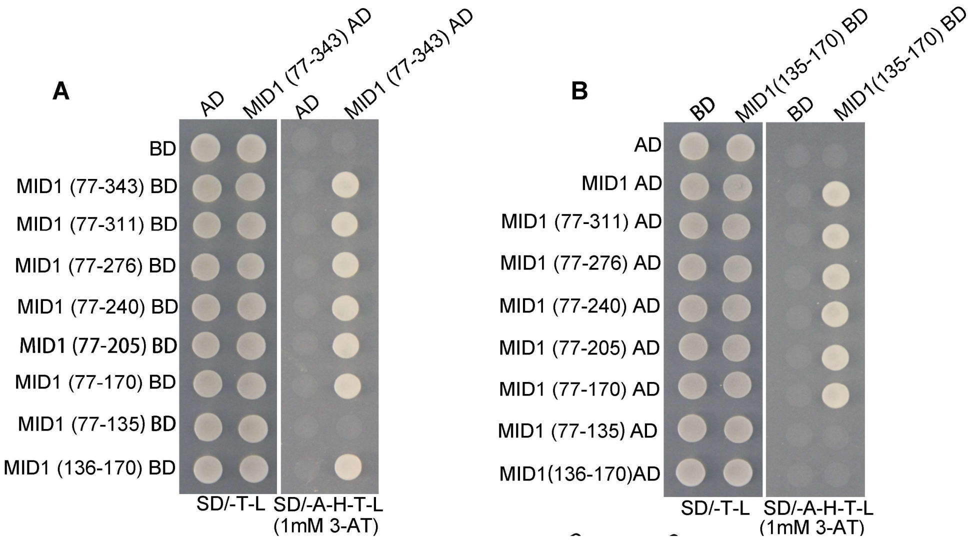

Supplemental Figure 8 Identification of Domains Necessary for MID1 Homodimer

(A) Identification of domains directly mediating the physical interaction during MID1 homodimer formation. 1mM 3-AT was added to inhibit the autoactivation. **(B)** Identification of domains that can facilitate the formation of MID1 homodimer. 1mM 3-AT was added to inhibit the autoactivation.

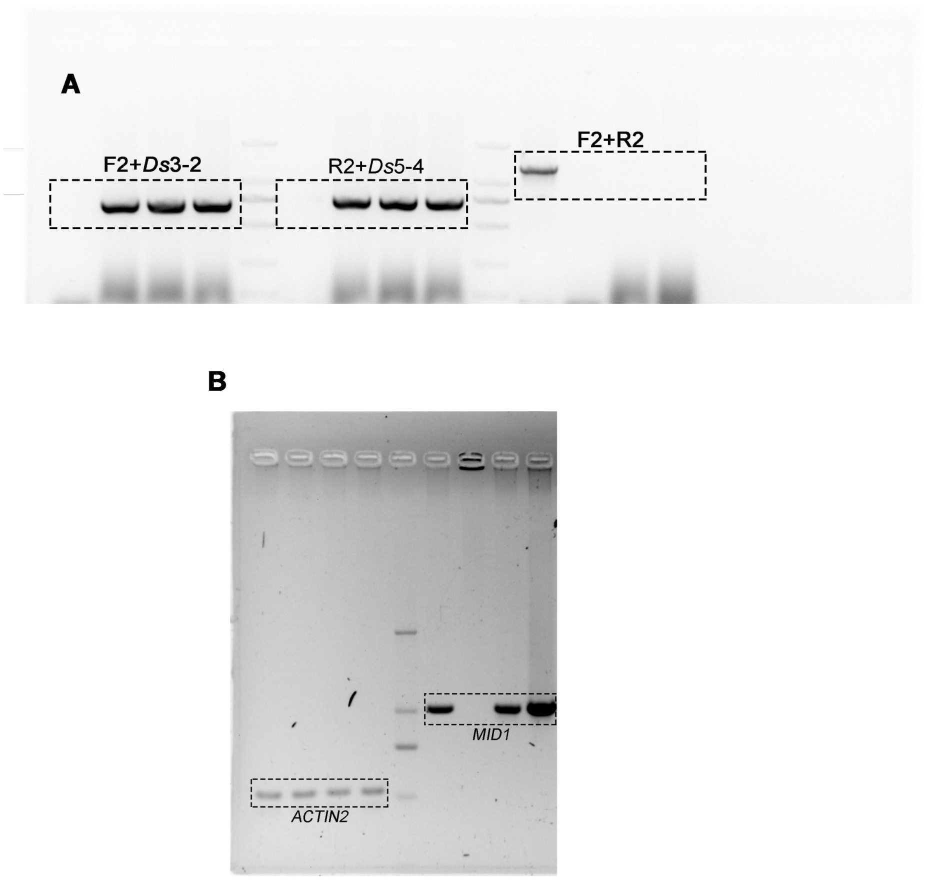

Supplemental Figure 9 Uncropped image of Figure 3C

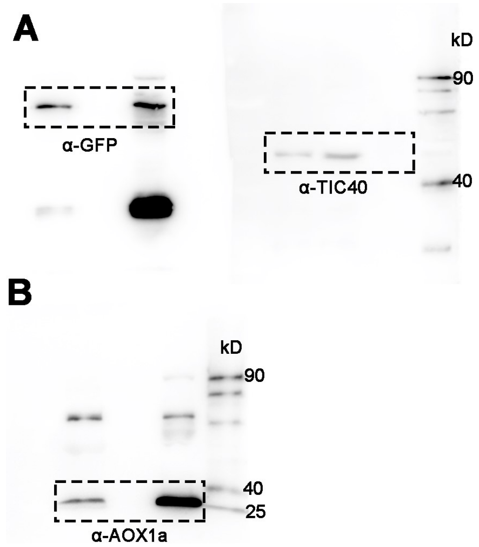

Supplemental Figure 10 Uncropped image of Figure 4D

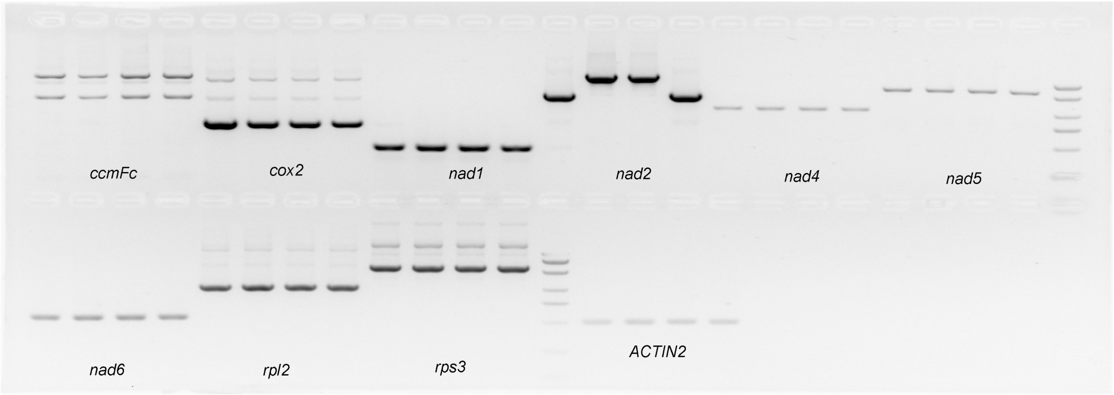

Supplemental Figure 11 Uncropped image of Figure 5B

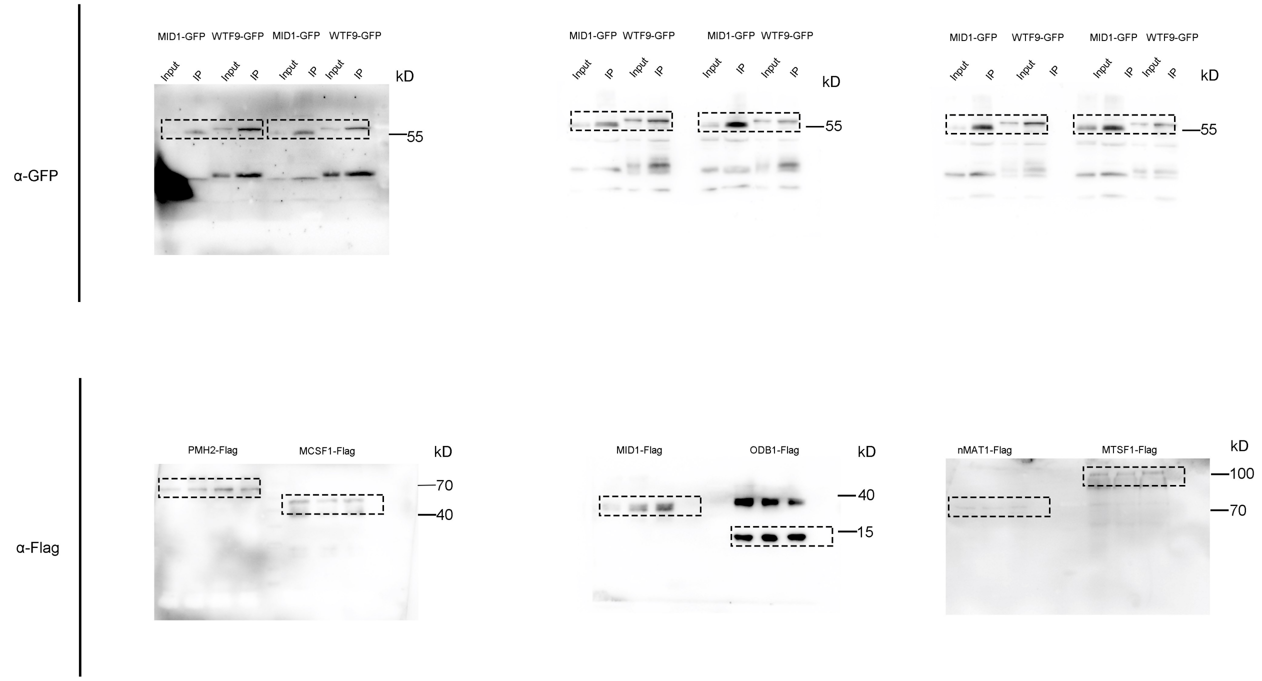

Supplemental Figure 12 Uncropped image of Figure 6B

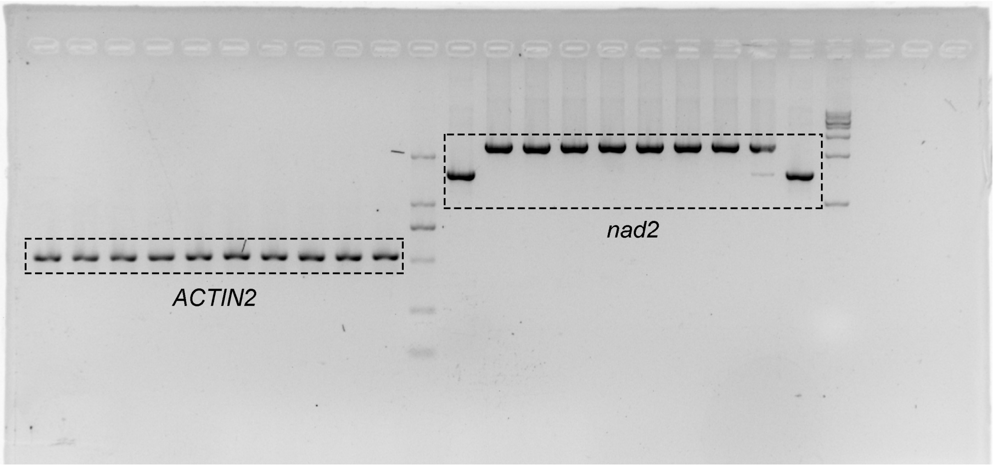

Supplemental Figure 13 Uncropped image of Figure 7C

	Primer name	Primer sequence (5'-3')
Primers for Y2H assay	MID1 BD 77aa forward	ATCTCAGAGGAGGACCTGCATCATAGAATCAGAGTCATTGACG
	MID1 BD 136aa forward	ATCTCAGAGGAGGACCTGCATCAGACTCATCTCCTTCTCTCT
	MID1 BD 135aa reverse	GAATTCGGCCTCCATGGCCATAGGGTTAGGTAGACAGCCAGAACG
	MID1 BD 170aa reverse	GAATTCGGCCTCCATGGCCATATCAGGACTGTATCCAATC
	MID1 BD 205aa reverse	GAATTCGGCCTCCATGGCCATATCAGGAATACAGCCAGCTGCAC
	MID1 BD 240aa reverse	GAATTCGGCCTCCATGGCCATTGGAGAGATCCCAGCTTTGCTCA
	MID1 BD 276aa reverse	GAATTCGGCCTCCATGGCCATCTCCACAGGGTAATCCCGAC
	MID1 BD 311aa reverse	GAATTCGGCCTCCATGGCCATATACGGTATGAACCCTC
	MID1 BD 343aa reverse	GAATTCGGCCTCCATGGCCATAGACCTCAGTTCAGAAACCCTTTG
	nMAT1 BD 90aa forward	ATCTCAGAGGAGGACCTGCATTGGGTCTTAGCTTATCAACGAAC
	nMAT1 BD 711aa reverse	GAATTCGGCCTCCATGGCCATTGTTTGTGAGTCTAATTTCTGCAACC
	MCSF1 BD 28aa forward	ATCTCAGAGGAGGACCTGCATTCACGACTCCGAGACCTTTACA
	MCSF1 BD 405aa reverse	GAATTCGGCCTCCATGGCCATGGTTGTCTCGTCAGGAGAATCT
	MTSF1 BD 43aa forward	ATCTCAGAGGAGGACCTGCATTCCACTCCTCCGCCGGATGACAT
	MTSF1 BD 997aa reverse	GAATTCGGCCTCCATGGCCATAGTCCCATCAGATGTTTTCTTTTC
	ODB1 BD 47aa forward	ATCTCAGAGGAGGACCTGCATACTTCAGGAATCAGTAGACCACTC
	ODB1 BD 176aa reverse	GAATTCGGCCTCCATGGCCATCAAAGCATCCTCGTGATAAAGATG
	PMH2 BD 26aa forward	ATCTCAGAGGAGGACCTGCATAATACTGTTCTGTTTCACAACCTGGC
	PMH2 BD 616aa forward	GAATTCGGCCTCCATGGCCATGTAAGATCTTTTCCCATCATTTGATC
	MID1 AD 77aa forward	GACGTACCAGATTACGCTCATCATAGAATCAGAGTCATTGACG
	MID1 AD 136aa forward	GACGTACCAGATTACGCTCATCAGACTCATCTCCTTCTCTCT
	MID1 AD 135aa reverse	TTCACTGGCCTCCATGGCCATAGGGTTAGGTAGACAGCCAGAACG
	MID1 AD 170aa reverse	TTCACTGGCCTCCATGGCCATATCAGGACTGTATCCAATC
	MID1 AD 205aa reverse	TTCACTGGCCTCCATGGCCATATCAGGAATACAGCCAGCTGCAC
	MID1 AD 240aa reverse	TTCACTGGCCTCCATGGCCATTGGAGAGATCCCAGCTTTGCTCA
	MID1 AD 276aa reverse	TTCACTGGCCTCCATGGCCATCTCCACAGGGTAATCCCGAC
	MID1 AD 311aa reverse	TTCACTGGCCTCCATGGCCATATACGGTATGAACCCTC
	MID1 AD 343aa reverse	TTCACTGGCCTCCATGGCCATAGACCTCAGTTCAGAAACCCTTTG
	nMAT1 AD 90aa forward	GACGTACCAGATTACGCTCATTGGGTCTTAGCTTATCAACGAAC
	nMAT1 AD 711aa reverse	TTCACTGGCCTCCATGGCCATTGTTTGTGAGTCTAATTTCTGCAACC
	MCSF1 AD 28aa forward	GACGTACCAGATTACGCTCATTCACGACTCCGAGACCTTTACA
	MCSF1 AD 405aa reverse	TTCACTGGCCTCCATGGCCATGGTTGTCTCGTCAGGAGAATCT
	MTSF1 AD 43aa forward	GACGTACCAGATTACGCTCATTCCACTCCTCCGCCGGATGACAT
	MTSF1 AD 997aa reverse	TTCACTGGCCTCCATGGCCATAGTCCCATCAGATGTTTTCTTTTC
	ODB1 AD 47aa forward	GACGTACCAGATTACGCTCATACTTCAGGAATCAGTAGACCACTC
	ODB1 AD 176aa reverse	TTCACTGGCCTCCATGGCCATCAAAGCATCCTCGTGATAAAGATG
	PMH2 AD 26aa forward	GACGTACCAGATTACGCTCATAATACTGTTCTGTTTCACAACCTGGC
	PMH2 AD 616aa forward	TTCACTGGCCTCCATGGCCATGTAAGATCTTTTCCCATCATTTGATC
Primers for protoplast transformation	MID1 GFP forward	ATCGAATTCCTGCAGCCCGGGATGGCAATTCGCTTGACGCATC
	MID1(90aa) GFP reverse	CACCATTCTAGAACTAGTAACAAAACTTTGGAGGATTTCGTC
	MID1 GFP reverse	CACCATTCTAGAACTAGTAGACCTCAGTTCAGAAACCCTTTG
	WTF9 forward	ATCGAATTCCTGCAGCCCGGGATGCTCTCTATTCGCCGCCATGCCAA

	WTF9 reverse	CACCATTCTAGAACTAGTGCCTTCAAAATCCAAATCCAAATC
	nMAT1 Flag forward	TCTCTCGAGGTCGACGGTATCGAATGAAAAGACTGACATATCCATTGAG
	nMAT1 Flag reverse	TTCTGCAGGAATTCGATATCTGTTTGTGAGTCTAATTTCTGCAACC
	MCSF1 Flag forward	TCTCTCGAGGTCGACGGTATCGAATGTTCTTGATTCGTCTCTCCCGC
	MCSF1 Flag reverse	TTCTGCAGGAATTCGATATCGGTTGTCTCGTCAGGAGAATCT
	MTSF1 Flag forward	TCTCTCGAGGTCGACGGTATCGAATGAACAAAACAGTCGTAAGATGTC
	MTSF1 Flag reverse	TTCTGCAGGAATTCGATATCAGTCCCATCAGATGTTTTCTTTTC
	ODB1 Flag forward	TCTCTCGAGGTCGACGGTATCGAATGGCGGGGTTAGGGTTA
	ODB1 Flag reverse	TTCTGCAGGAATTCGATATCCAAAGCATCCTCGTGATAAAGATG
	PMH2 Flag forward	TCTCTCGAGGTCGACGGTATCGAATGATCACTACAGTGCTACGACG
	PMH2 Flag reverse	TTCTGCAGGAATTCGATATCGTAAGATCTTTTCCCATCATTTGATC
Primers for genetic complmentation	MID1 forward	GGGGGACTCTAGAGGATCCCCATGGCAATTCGCTTGACGCATC
	MID1 135aa reverse	CTCGCCCTTGCTCACGGTACCCAGGGTTAGGTAGACAGCCAGAACG
	MID1 170aa reverse	CTCGCCCTTGCTCACGGTACCCATCAGGACTGTATCCAATC
	MID1 205aa reverse	CTCGCCCTTGCTCACGGTACCCATCAGGAATACAGCCAGCTGCAC
	MID1 240aa reverse	CTCGCCCTTGCTCACGGTACCCTGGAGAGATCCCAGCTTTGCTCA
	MID1 276aa reverse	CTCGCCCTTGCTCACGGTACCCCTCCACAGGGTAATCCCGAC
	MID1 311aa reverse	CTCGCCCTTGCTCACGGTACCCATACGGTATGAACCCTC
	MID1 343aa reverse	CTCGCCCTTGCTCACGGTACCCAGACCTCAGTTCAGAAACCCTTTG
	MID1 Δ(101-135aa) inter reverse	GAGAGAAGGAGATGAGTCTGAATCTTGGGCAGTGAACGA
	MID1 Δ(101-135aa) inter forward	TCGTTCACTGCCCAAGATTCAGACTCATCTCCTTCTCTC
	MID1 Δ(136-170aa) inter reverse	AAGTAATTGCAGGTTCCAGTAGGGTTAGGTAGACAGCCAG
	MID1 Δ(136-170aa) inter forward	CTGGCTGTCTACCTAACCCTACTGGAACCTGCAATTACTT
	MID1Pro forward	CAGTGCCAAGCTTGCATGCCGTAACTATAATGTTTGACGG
	MID1Pro reverse	TGCGTCAAGCGAATTGCCATTTCCTATTCTCAGAGACCTG
	MID1 CDS forward	CAGGTCTCTGAGAATAGGAAATGGCAATTCGCTTGACGCA
	MID1 Flag reverse	GTCGACAGGCCTTTCGAAAGACCTCAGTTCAGAAACCC
	MID1 GUS reverse	TTCGAGCTCGGTACCCGGGGATCCAGACCTCAGTTCAGAAACCC
Primers for RT-PCR	F1	ATGGCAATTCGCTTGACGCATCT
	R1	CTAAGACCTCAGTTCAGAAACCC
	F2	CCGGATTATTCCTCCTGGGATTAG
	R2	CCAGGATCGTAACTGCAGCTATTC
	ACTIN2 RT forward	ATCCTTCCTGATATCGAC
	ACTIN2 RT reverse	GAGAAGATGACTCAGATC
	ccmFc forward	ATTTTTATGGTCGTGCCTTGTGGCA
	ccmFc reverse	TGAACTCCACGGAACTTTCTCGATT
	cox2 forward	CCTCACAATTTCTCCTTGTGATGC
	cox2 reverse	TTCCCCGGTTTGGGGGATTAAT
	rpl2 forward	TGAGACCAGGGAGAGCAAGAGC
	rpl2 reverse	ACAGTGAATAAGGGCTTAGGATGG
	rps3 forward	ATGGCACGAAAAGGAAATCCG
	rps3 reverse	TCGTACGTTTCGGATATAGCAC

Primers for RT-PCR analysis of *nad* transcripts are from des Francs-Small *et al*., 2014

Primers for qRT-PCR	MID1 qRT forward	TTTTTCCGGTCAATCTCGTC
	MID1 qRT reverse	GCAGTGAACGAGGTCTCACA
	ACTIN2 qRT forward	ACACTGTGCCAATCTACGAGGGTT
	ACTIN2 qRT reverse	ACAATTTCCCGCTCTGCTGTTGTG
Primers for luciferase complementation assay	MID1-NLuc forward	AGAACACGGGGGACGAGCTCGGTACCATGCATAGAATCAGAGTCATTGACG
	MID1-NLuc reverse	AGTCCATTTGTTGGATCCCGGGTACCAGACCTCAGTTCAGAAACCCTTTGTC
	WTF9-NLuc forward	AGAACACGGGGGACGAGCTCGGTACCATGCACTTCTTAAGGAAGTTTCCTTCAA
	WTF9-NLuc reverse	AGTCCATTTGTTGGATCCCGGGTACCGCCTTCAAAATCCAAATCCAAATCT
	CLuc-MID1 forward	TCTCGTACGCGTCCCGGGGCGGTACCCATAGAATCAGAGTCATTGACG
	CLuc-MID1 reverse	AGTCCATTTGTTGGATCCCGGGTACCCTAAGACCTCAGTTCAGAAACCCT
	CLuc-MTSF1 forward	TCTCGTACGCGTCCCGGGGCGGTACCTCCACTCCTCCGCCGGATGACATGT
	CLuc-MTSF1 reverse	AGTCCATTTGTTGGATCCCGGGTACCCTAAGTCCCATCAGATGTTTTC
